# Deciphering the Translation Initiation Factor 5A Modification Pathway in Halophilic Archaea

**DOI:** 10.1155/2016/7316725

**Published:** 2016-12-08

**Authors:** Laurence Prunetti, Michael Graf, Ian K. Blaby, Lauri Peil, Andrea M. Makkay, Agata L. Starosta, R. Thane Papke, Tairo Oshima, Daniel N. Wilson, Valérie de Crécy-Lagard

**Affiliations:** ^1^Department of Microbiology and Cell Science, Institute for Food and Agricultural Sciences and Genetic Institute, University of Florida, P.O. Box 110700, Gainesville, FL 32611-0700, USA; ^2^Gene Center and Department for Biochemistry, University of Munich, 81377 Munich, Germany; ^3^Center for Integrated Protein Science Munich, University of Munich, 81377 Munich, Germany; ^4^Faculty of Science and Technology, Institute of Technology, University of Tartu, Tartu, Estonia; ^5^Department of Molecular and Cell Biology, University of Connecticut, 91 N. Eagleville Rd, Storrs, CT 06269, USA; ^6^Institute of Environmental Microbiology, Kyowa Kako Co. Ltd., Tadao 2-15-5, Machida 194-0035, Japan

## Abstract

Translation initiation factor 5A (IF5A) is essential and highly conserved in Eukarya (eIF5A) and Archaea (aIF5A). The activity of IF5A requires hypusine, a posttranslational modification synthesized in Eukarya from the polyamine precursor spermidine. Intracellular polyamine analyses revealed that agmatine and cadaverine were the main polyamines produced in* Haloferax volcanii* in minimal medium, raising the question of how hypusine is synthesized in this halophilic Archaea. Metabolic reconstruction led to a tentative picture of polyamine metabolism and aIF5A modification in* Hfx. volcanii *that was experimentally tested. Analysis of aIF5A from* Hfx. volcanii* by LC-MS/MS revealed it was exclusively deoxyhypusinylated. Genetic studies confirmed the role of the predicted arginine decarboxylase gene* (HVO_1958)* in agmatine synthesis. The agmatinase-like gene* (HVO_2299)* was found to be essential, consistent with a role in aIF5A modification predicted by physical clustering evidence. Recombinant deoxyhypusine synthase (DHS) from* S. cerevisiae* was shown to transfer 4-aminobutyl moiety from spermidine to aIF5A from* Hfx. volcanii in vitro. *However, at least under conditions tested, this transfer was not observed with the* Hfx. volcanii* DHS. Furthermore, the growth of* Hfx. volcanii* was not inhibited by the classical DHS inhibitor GC7. We propose a model of deoxyhypusine synthesis in* Hfx. volcanii* that differs from the canonical eukaryotic pathway, paving the way for further studies.

## 1. Introduction

The translation initiation factor 5A (IF5A) is highly conserved in Eukaryotes (eIF5A) and Archaea (aIF5A), whereas bacteria harbor the homolog elongation factor P (EF-P). IF5A performs multiple intracellular functions and is involved in cell growth and death [1, 2]. While both eIF5A and EF-P proteins had been initially linked to translation initiation [1, 3], recent studies have shown that they are required for the efficient translation of proteins containing polyproline stretches (Pro-Pro-Pro; Pro-Pro-Gly) [4–10].

Major differences between IF5A and EF-P exist, even if their core function in translation is conserved. First, both eIF5A and aIF5A are essential [11, 12] whereas deletion of bacterial* efp* can be viable and leads to a range of phenotypes depending on the organism [13–16]. Second, the posttranslational modification of a strictly conserved lysine (K50, Human eIF5A) into pN^*ε*^-(4-amino-2hydroxybutyl)-lysine or hypusine is required for eIF5A activity and the hypusine modification pathway is conserved in Eukaryotes [11]. Conversely, hypusine is not found in the bacterial EF-P proteins where the equivalent lysine can be modified by the addition of a *β*-lysine residue [17–21] or by rhamnosylation [22, 23].

The eukaryotic hypusine synthesis pathway contains two consecutive steps [11, 24–27]. The first enzyme, deoxyhypusine synthase (DHS), catalyzes the transfer of the 4-aminobutyl moiety of spermidine to the target lysine residue forming the deoxyhypusine intermediate [26]. This intermediate is then hydroxylated by deoxyhypusine hydroxylase (DOHH) to form the biologically active hypusinylated factor [11]. N^1^-Guanyl-1,7-diaminoheptane (GC7), a spermidine homolog, very efficiently inhibits the first step of hypusination by binding to DHS [28, 29]. In Eukaryotes, the hypusine modification of eIF5A occurs shortly after the synthesis of eIF5A and no pool of unmodified proteins has ever been detected [30, 31]. Interestingly, although the deoxyhypusine/hypusine modification is essential in all eukaryotes, only DHS is essential in* Saccharomyces cerevisiae* and eIF5A partially modified with deoxyhypusine is functional [1, 3].

The archaeal aIF5A proteins and their modification pathways are poorly characterized. DHS homologs are present in all sequenced archaeal genomes; however to date, no DOHH orthologue has been identified in any archaeal genomes or proteomes [25, 26], raising questions about the nature of this final modification in Archaea. Early analyses based on amino acid composition data reported the presence of both hypusine and deoxyhypusine in Archaea [32]. Hypusine was detected in several Crenarchaea like* Sulfolobus acidocaldarius*,* Pyrodictium occultum*,* Thermoproteus tenax*, and* Acidianus ambivalens*. However, high levels of deoxyhypusine but no (or only low levels) traces of hypusine were found in Euryarchaeota (i.e., halobacteriales, methanogen, thermococcales, and thermoplasmales) [32] and the specific nature of the modification found in aIF5A proteins was never confirmed by mass-spectrometry (MS) methods. Growth inhibition by GC7 has been reported in four archaeal species* S. acidocaldarius*,* Sulfolobus solfataricus*,* Halobacterium halobium* DSM 670, and* Haloferax mediterranei* DSM1411 [33], suggesting that the archaeal deoxyhypusine pathway is essential, as in eukaryotes.* S. acidocaldarius* aIF5A is to date the only archaeal protein for which the presence of the hypusine modification has been experimentally confirmed by amino acid composition [34]. The presence of the DHS encoding genes in archaeal genomes, combined with the GC7 inhibition results, strongly suggests that deoxyhypusine is synthesized by similar mechanisms in Archaea and Eukarya, yet many questions remain.

Spermidine is the 4-aminobutyl donor for the eukaryotic DHS enzyme [11] but the great diversity of polyamines found in Archaea suggests this might not always be the case in this kingdom of life. Indeed, spermidine was detected in* Thermococcus kodakarensis* [35] and in various* Sulfolobus *species [36] and homospermidine (that could also be an aminobutyl donor for DHS [37]) was found to be an abundant polyamine in methanogens [38] (Table S1). However, the composition of intracellular polyamines was analyzed in 117 archaeal halophiles strains and trace amounts of spermidine and/or spermine were detected in only 20 strains [39]. Agmatine appears to be the major accumulating polyamine in this order (Table S1 in Supplementary Material available online at http://dx.doi.org/10.1155/2016/7316725) [36, 40, 41]. Agmatine is the precursor of agmatidine, an essential modification of the anticodon wobble cytosine in archaeal tRNA^Ile^_CAU_ [42–44]. Agmatine is therefore an essential archaeal metabolite that can be either synthesized* de novo* or salvaged [42]. More generally, while archaeal polyamine metabolic pathways have been partially elucidated in thermophilic Archaea [35, 45], little is known about polyamine pathway in halophilic Archaea.

By combining metabolic reconstruction, genetics, comparative genomics, and biochemical studies, we set out to elucidate both the polyamine and aIF5A modification pathways in the model halophile* Haloferax volcanii*.

## 2. Experimental Procedures

### 2.1. Strains and Growth Conditions

All strains, plasmids, and oligonucleotides used in this study are listed in Tables S2 and Table S3.* Hfx. volcanii* H26 was used as the parent strain.* Escherichia coli* derivatives were routinely grown in LB-Lennox (LB) (Fisher) or LB agar (Fisher) at 37°C and supplemented when required with ampicillin (Amp, 100 *μ*g/mL).* Hfx. volcanii* strains were grown at 42°C in either rich (ATCC 974) or minimal media (Hv_min) as previously described [46]. Novobiocin (0.1 *μ*g/mL) and agar (5 g/L) were included as needed. Uracil was dissolved to 50 mg/mL in 100% (v/v) DMSO and added to growth medium at a final concentration of 50 *μ*g/mL. Transformations of* E. coli* and* Hfx. volcanii* were performed as previously described [46].

### 2.2. Plasmid and Strain Constructions

#### 2.2.1. *HVO_1958* and* HVO_2299* Deletions

Plasmids used to delete the* HVO_1958* and* HVO_2299 *genes were constructed as described previously [44]. Briefly, ~600 bp regions up- and downstream of* HVO_1958 *and* HVO_2299 *were PCR-amplified from purified genomic DNA using Phusion polymerase (NEB) and the oligonucleotides listed in Table S3 and then inserted using In-Fusion (Clontech) into pTA131, linearized by digestion with* Eco*RI and* Xho*I. The resulting plasmids, pIKB313 and pIKB298, containing the deletion constructs for* HVO_1958* and* HVO_2299, *respectively, were validated by sequencing before passaging through* E. coli* Inv110 (Invitrogen). The plasmids were subsequently transformed into* Hfx. volcanii* strain H26, and the deletion strains were generated by the* pyrE2-*based “pop-in/pop-out” deletion method [47, 48], with standard media preparations [46] except that the media was supplemented with 100 *μ*M agmatine for deletion of* HVO_1958* or with or without 1 mM putrescine for the deletion of* HVO_2299*. Deletion of* HVO_1958* was validated by PCR methods using the primers ext f and ext r (Table S3), generating strain VDC3253 (Table S2).

### 2.3. LSP5061

H26 was transformed with pIKB298 and the pop-in was generated [47, 48] with standard media preparation. Ten isolated colonies of the pop-in were pooled together and grown in liquid to generate competent cells [47]. The competent “pop-in strain” was transformed with the pLSP21 plasmid that was constructed by cloning* HVO_2299 *under the control of the P_TNA_ promoter into the pJAM202 derivative pPT002 (Table S2) in the presence of 2.5 mM tryptophan and novobiocin (0.1 *μ*g/mL) before generating the pop-out (LSP5061) in presence of 2.5 mM tryptophan and novobiocin (0.1 *μ*g/mL) [49]. Deletion of* HVO_2299 *from the* Hfx. volcanii* genome was validated by PCR methods using the primers FW-391 and RV-391 (Table S3).

#### 2.3.1. TIF5A-C-Term His Integrant

VDC2577, containing the native copy of* aIF5A (HVO_2300)* with a C-terminal His-tag (6x His), was generated by transformation of* Hfx. volcanii* H26 with pIKB473 (Table S2), constructed as described in previously [44], with the oligonucleotides listed in Table S3.

### 2.4. Physiology Studies

#### 2.4.1. Stress Conditions

Cells were subjected to different stress conditions as described in [50]. Briefly, H26 were inoculated into 5 mL ATCC 974 and then diluted into 25 mL of fresh ATCC 974 at OD_600_ of 0.0074. The cells were grown at 42°C for 24 hours in order to reach early exponential growth phase (OD_600_ = 1.29 ± 0.049) before the stress was applied for 4 hours (oxidative and cold shock) or 24 hours (proteasome inhibitor). Oxidative stress was induced by the addition of H_2_O_2_ to a final concentration of 0.78% (w/v) for 4 hours at 42°C with shaking (200 rpm). For cold shock, the cells were grown at 30°C for 4 hours with shaking (200 rpm). The proteasome inhibitor, bortezomib (LC Laboratories), was added at 100 *μ*M final concentration as described in [51]. Cultures were incubated with shaking (200 rpm) at 42°C for 24 hours. After the stress, growth was monitored and 2 mL of culture at an OD_600_ 0.043 was harvested for immunoblot analysis.

#### 2.4.2. Culture in Presence of GC7

To monitor the effect of N^1^-guanyl-1,7-diaminoheptane (GC7) (Santa Cruz Biotechnologies) on* Hfx. volcanii *growth, H26 was inoculated into 5 mL of ATCC 974 medium and grown to log phase. Cells were subcultured into 5 mL of fresh Hv_min medium until exponential phase. The cells (1 mL at an OD_600_ = 1) were washed 4 times in Hv_min medium and serial dilutions in Hv_min medium were spot-plated (15 *μ*L) onto solid Hv_min agar in the absence or presence of 1 mM GC7. The cells were grown at 45°C for 4 days. The experiments were done with two biological replicates (i.e., two independent cultures) and three technical duplicates (three measurements per culture).

#### 2.4.3. Culture in Presence of Agmatine

*Hfx. volcanii* H26 (WT, parent) and Δ*adc* (VDC3253) were grown in 5 mL ATCC 974 medium supplemented with mM agmatine at 42°C. Cells were then subcultured into 5 mL Hv_min medium at 42°C. Cells grown to stationary phase (after 48 hours at 42°C) were washed 4 times in Hv_min medium. Cell density was normalized by dilution to OD_600_ of 1 and serial dilutions were spot-plated (15 *μ*L) on solid agar Hv_min medium in the absence or presence of agmatine (1 *μ*M or 5 *μ*M). Each experiment was performed with two biological replicates and three technical replicates.

### 2.5. Polyamines Analyses

#### 2.5.1. Sample Preparation

H26 was grown in 1 L of Hv_min medium in 2.8 L Fernbach flasks at 42°C (200 rpm). At different points of growth (early exponential OD_600_ = 0.0805, mid-exponential OD_600_ = 0.86, and late exponential OD_600_ = 1.2), cells were harvested at 5,500 rpm (SLA 3000, Sorvall) for 40 min at 4°C and then washed with 50 mM HEPES, 2 M NaCl, and pH 8 buffer. The pellets were frozen in liquid nitrogen prior to desiccation. Experiments were done with two biological replicates.

#### 2.5.2. Polyamines Extraction

An appropriate amount of the cells (between 7 and 110 mg) was transferred into an Eppendorf tube and suspended in 1 mL of 10% trichloroacetic acid solution. The suspension was shaken for 1 min repeatedly and kept at room temperature overnight. The suspension was then centrifuged and the supernatant was filtered using 0.2 *μ*m membrane filter in order to remove fine dust. The filtrate was diluted in water if necessary and applied on a polyamine analyzer.

#### 2.5.3. Polyamines Analysis

Polyamines were analyzed with a CK10S column (8.0 × 70 mm, Mitsubishi Kasei) as described previously [52]. Briefly, the column was developed with Type 2 buffer (citrate buffer containing 125 g/L of KCl) at 60°C, at a flow rate of 2 mL/min. Polyamines were detected using* o-*phthalaldehyde and fluorescence at *λ*450 nm (excitation at *λ*340 nm) was recorded. The recorded chart was analyzed as described previously [52]. For each biological sample, two technical replicates were analyzed. The standard used was a mixture of 2.0 *μ*M agmatine and 2.8 *μ*M cadaverine.

### 2.6. Protein Purification

#### 2.6.1. aIF5A-His-C-Term Purification

*E. coli *Rosetta gami 2 (DE3) (Novagen) strains freshly transformed with plasmid pLSP24 were grown in 1 L LB Amp in 2.8 L Fernbach flasks at 37°C (200 rpm) in the presence of 2% glucose. Isopropyl *β*-D-1-thiogalactopyranoside (IPTG) was added to a final concentration of 0.4 mM at log phase (OD_600_ of 0.4–0.6 units), and cultures were shifted to 25°C for 3 hours (200 rpm) prior to harvest. Cells were harvested by centrifugation (5,000 ×g, 4°C, 15 min) and stored at −80°C. Cells (10-gram wet weight) were resuspended in 20–25 mL of 20 mM Tris-HCl pH 8 and 2 M NaCl (buffer A) and broken by passage three times by French Press at a pressure of 2000 psi. After 15 min of centrifugation at 3,000 ×g (4°C) to remove unbroken cells, the supernatant was filtered through a 0.8 *μ*M cellulose acetate membrane (Fisher Scientific, USA). The supernatant was then applied to a HisTrap HP column (1 mL, GE Healthcare) preequilibrated with buffer A with 40 mM imidazole and washed with the same buffer at room temperature. aIF5A-C-term His-tag protein was eluted from the column with 20 mM Tris-HCl pH 8, 2 M NaCl, and 500 mM imidazole (buffer B). Purified proteins were detected by Western blotting and/or Coomassie Blue R-250 staining after separation by 10% SDS-PAGE. The purified proteins were precipitated by trichloroacetic acid (TCA) as described by Sanchez [53] before loading on SDS-PAGE.

#### 2.6.2. T7-His-DHS

*Hfx. volcanii* LSP5021 (H26 carrying pLSP23) was used for the purification of T7-His-DHS. LSP5021 (40-gram wet weight) were resuspended in 80 mL of buffer A and broken by passage three times through a French Press at a pressure of 2000 psi as described before. Briefly, the supernatant was applied to a HisTrap HP column (5 mL, GE Healthcare). Then the fraction was dialyzed overnight against 1 L of buffer A. The sample was applied to a hydroxyapatite column (HTP) (25 mL) preequilibrated with buffer A at flow rate of 1 mL/min and eluted with a continuous gradient of Tris-HCl 20 mM, 2 M NaCl, and 400 mM sodium phosphate pH 8. Fractions of 1 mL were collected. The T7-His-DHS was eluted from the column with 40 mM of sodium phosphate in buffer A. The eluate was concentrated (Vivaspin 3,000 Dalton molecular weight cut off, Sartorius Stedim Biotech) and applied to a Sephacryl S-200 HR gel filtration column equilibrated with buffer A at a flow rate of 0.3 mL/min. Fractions of 1 mL were collected.

#### 2.6.3. Purification of IF5A and DHS from* Saccharomyces cerevisiae* and* Thermococcus kodakarensis*

The coding sequences of the respective proteins were cloned into pET28a (+) vector with an additional (6x) His-tag and TEV cleavage site using oligo pairs depicted in the supplemental data (Table S3). Proteins were overexpressed in BL21* E. coli *cells grown at 37°C from overnight culture in LB in presence of 50 *μ*g/mL kanamycin. Protein expression was induced at OD_600_ of 0.4 with a final concentration of 1 mM IPTG (Roth). After 1 hour of expression cells were lysed using a microfluidizer (Microfluidizer Processor Microfluidics Newton, USA). The cell lysate was cleared using a SS34 rotor (Sorvall) at 4°C and 44,100 ×g for 30 min. Purification of His-tagged proteins was done with Protino Ni-NTA agarose beads (Macherey-Nagel). The final eluate was applied onto a Superdex HiLoad S75 16/60 column (GE Healthcare) to yield the final concentrated protein in gel filtration buffer (50 mM HEPES pH 7.4, 50 mM KCl, 100 mM NaCl, and 5 mM 2-mercaptoethanol). 

### 2.7. Deoxyhypusine Synthase Assay

#### 2.7.1. *Thermococcus kodakarensis*

DHS modification assay was performed in presence of either 75 *μ*M radioactive [^14^C] spermidine trihydrochloride (GE Healthcare) or 3 *μ*M putrescine dihydrochloride [1,4-^3^H(N)] (Perkin Elmer). The reaction mixture contained the substrates 5 *μ*M of* S. cerevisiae* eIF5A or* T. kodakarensis* aIF5A, as well as 2 mM nicotinamide adenine dinucleotide (NAD^+^), 2.5 *μ*M* S. cerevisiae* DHS, or* T. kodakarensis* DHS in 0.2 M glycine-NaOH buffer, pH 9.4 (total volume 30 *μ*L). After 120 minutes of incubation at 37°C, incorporation of radiolabeled deoxyhypusine was monitored using a 16.5% Tricine-PAGE gel, which was subsequently dried and exposed to high-performance autoradiography films (GE Healthcare). Color Prestained Broad Range Protein Standard (NEB) was used as molecular weight marker.

#### 2.7.2. *Haloferax volcanii*

DHS was tested in cell extract overexpressing DHS. For DHS assay, the experiment was conducted in presence of either 75 *μ*M radioactive [^14^C] spermidine trihydrochloride (111 mCi/mmol, GE Healthcare) or 3 *μ*M putrescine dihydrochloride [1,4-^3^H(N)] (62 Ci/mmol, Perkin Elmer). Each 30 *μ*L sample contained 20 *μ*L of* Hfx. volcanii* extract (overexpressing DHS at a concentration of 3 mg/mL), 5 *μ*L of 1.5 mg/mL* Hfx. volcanii* aIF5A, 1 *μ*L of 100 mM nicotinamide adenine dinucleotide (NAD+), and the respective substrate. All samples were incubated at 42°C for 2 hours. Due to the presence of high salt concentrations in the* Hfx. volcanii* extract all samples were subjected to buffer exchange (50 mM HEPES, 100 mM K(OAc) and 25 mM Mg(OAc))* via* Amicon Ultra 0.5 mL Ultracel 3 K (Merck Millipore) before gel electrophoresis. The final samples were separated on a 16.5% Tricine gel. For evaluation of the protein size Spectra Multicolor Low Range Protein Ladder (Thermo Scientific) was used. Dried gels were exposed to high-performance autoradiography films (GE Healthcare) to evaluate the occurrence of aIF5A modification by DHS in the respective sample. The control samples with* S. cerevisiae* DHS were prepared as described for the* T. kodakarensis *DHS assay. To test the activity of* Hfx. volcanii* T7-His-DHS, 200 pmol of radioactive polyamines (agmatine, putrescine, or spermidine) was mixed with 20 pmol of T7-His-DHS in the presence or absence of 100 pmol of* Hfx. volcanii* aIF5A in 0.3 M glycine-NaOH pH 9.0, 2 M NaCl, and 1 mM NAD for 1 hour at 40°C.

#### 2.7.3. Synthesis of Radiolabeled Agmatine

Since radiolabeled agmatine is not available commercially, it was synthetized from [^14^C] arginine (Perkin Elmer) using arginine decarboxylase (SpeA). The plasmid pAS1 (carrying* speA-C-term-His*) was transformed into BL21* E. coli s*train (Novagen) and expressed in LB media. Cultures (200 mL) were induced with 1 mM IPTG at OD_600_ = 0.6 and grown for another 3 hours at 30°C. Thirty minutes prior to induction, chaperons were induced by addition of 1% (final concentration) of ethanol into LB media. Protein was purified using Ni-NTA spin columns (Qiagen) according to the manufacturer protocol, followed by buffer exchange (into 50 mM HEPES pH 7.4, 10 mM MgCl_2_, 100 mM NaCl, 50 mM KCl, 5 mM beta-mercaptoethanol, and 2% glycerol) using PD10 desalting columns (GE Healthcare). Agmatine synthesis from [^14^C] arginine (124 dpm/pmol) by SpeA was carried out in 0.3 M glycine-NaOH pH 9.0, 10 mM MgCl_2_, and 1 mM PLP at 37°C for 1 hour. The progress of the reaction was monitored using Silica Plates (Merck Millipore) developed in butanol/acetic acid/pyridine/formaldehyde 3 : 3 : 2 : 1 and exposed to high-performance autoradiography films (GE Healthcare).

### 2.8. Analytical Procedures

#### 2.8.1. Protein Concentration

Protein concentrations were determined by the bicinchoninic acid method [54] (Thermo Scientific Pierce BCA Protein Assay Kit, Rockford, IL) with bovine serum albumin (BioRad) as the protein standard.

#### 2.8.2. Electrophoresis

Protein samples were mixed in equal volume ratio with 2x loading buffer (containing 125 mM Tris-HCL pH 6.8, 20 mM *β*-mercaptoethanol, 4% (w/v) SDS, 20% (v/v) glycerol, and 0.01% (w/v) bromophenol blue) and boiled for 15 min. Samples were separated by 12% SDS-PAGE or gradient gel 4–15% (Biorad), using a mini-Protean III cell electrophoresis apparatus (Biorad) at 20 mA constant current at room temperature in a running buffer of 25 mM Tris and 190 mM glycine at pH 8.3 with 0.1% (w/v) SDS. After migration, proteins were stained in-gel with Coomassie Blue R-250 or were detected by Western blotting.

#### 2.8.3. Western Blotting

For aIF5A analysis during the growth in ATCC at 42°C, H26 cells (2 mL at OD 0.043 of the culture) were harvested via centrifugation (14,000 ×g, 10 min, 25°C) and treated as described before. Proteins were separated by SDS-PAGE (gradient gel 4–15%, Biorad) and transferred onto PVDF membrane (GE Healthcare). Equivalent protein loading was based on OD_600_ of cell culture (0.086 units per lane) and confirmed by staining parallel gels with Coomassie Blue. aIF5A protein was detected via immunoblot using TIF5A2 (1/10,000) or TIF5A3 (1/5,000), followed by a secondary anti-rabbit IgG antibody [HRP] (1/5,000) (GenScript, USA). The rabbit polyclonal antibodies TIF5A2 and TIF5A3 were produced using, respectively, the synthetic peptides CEIEYLEYEGQRKIV and MAKEQKQVRELQEGC (GenScript, USA). Membranes were visualized* via* chemiluminescence using Clarity western ECL (Biorad) with X-ray film (Fisher). aIF5A C-term His was detected with the anti C-term-His-HRP (Invitrogen) at a dilution 1/5,000. The deoxyhypusine/hypusine modification was detected with the antibody IU-88 (1/1,500) as described by [55].

### 2.9. Mass Spectrometry

Briefly, aIF5A was in-gel digested with LysC (Wako) according to the protocol of Shevchenko et al. [56] and desalted on C18-StageTips [57]. Peptides were then separated by reversed-phase liquid chromatography (LC) on a Dionex UltiMate RSLCnano 3000 system (Thermo Fisher Scientific). LC conditions were as follows: 30 min 3–40% B gradient (A: 0.1% formic acid, B: 0.1% formic acid/80% acetonitrile) at a flow rate of 200 nL/min, using 75 *μ*m × 300 mm fused silica emitter (New Objective, USA) packed in-house with Reprosil-Pur C18-AQ 3 *μ*m particles (Dr. Maisch, Germany). Eluted peptides were sprayed directly into a Q Exactive mass-spectrometer (Thermo Fisher Scientific) operated in data-dependent mode with up to ten MS/MS scans (NCE = 25) being recorded for each precursor ion scan. Peak lists for database search were prepared by using MSConvert. Database search was performed with the Mascot 2.3 search engine (Matrix Science) against the protein sequence database containing aIF5A sequence and common contaminant proteins, such as trypsin and keratins. Search parameters were 5 ppm for precursor mass tolerance, 0.02 Da for Orbitrap MS/MS mass tolerance, and up to three missed cleavages plus a number of variable modifications such as oxidation (M), hypusinylation (K), and deoxyhypusinylation (K). Peptide hits returned by Mascot were manually validated and annotated using xiSPEC (http://spectrumviewer.org/).

### 2.10. Bioinformatics

The gene sequences were obtained from NCBI. The BLAST tools [58] and resources at NCBI (https://www.ncbi.nlm.nih.gov/) were used routinely. Multiple sequence alignments were built using Clustal Omega [59] or MultAlin [60]. Protein domain analysis was performed using the Pfam database tools [61]. Transmembrane helices were predicted with the TMHMM Server v. 2.0 (http://www.cbs.dtu.dk/services/TMHMM-2.0/) [62]. Analysis of phylogenetic distribution and physical clustering was performed with STRING (http://string-db.org/.) [63] and the SEED Database [64].

## 3. Results and Discussion

### 3.1. Metabolic Reconstruction of Polyamines Metabolism and aIF5A Modification in* Hfx. volcanii*

**Figure 1:**
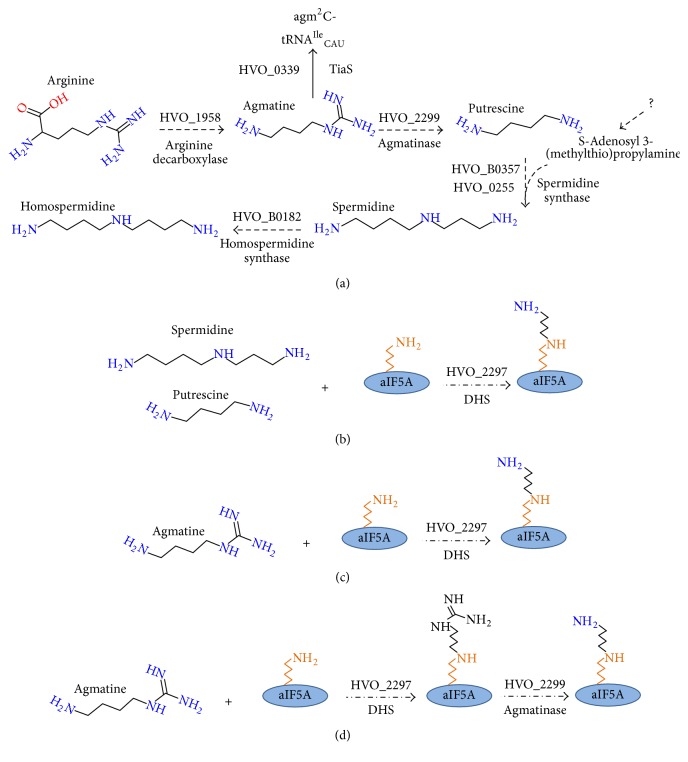
Proposed models of polyamines metabolism and aIF5A in* Hfx. volcanii*. (a) Predicted pathways of polyamines synthesis from arginine substrate. Dashed arrows indicate proposed reaction; solid arrow indicates known reaction. (b–d) Proposed substrates of DHS.* HVO_1958* encodes a potential arginine decarboxylase;* HVO_2299* an agmatinase-like;* HVO_0255* a spermidine synthase;* HVO_B0182* a spermidine synthase or a homospermidine synthase; and* HVO_2297* a potential deoxyhypusine synthase.

Spermidine is the donor for the eukaryotic DHS enzyme [11]. In a recent review on polyamine metabolism in all kingdoms of life [37], a core starter pathway for polyamine synthesis in Archaea through the decarboxylation of arginine by arginine decarboxylase (ADC) was proposed. Metabolic reconstruction in* Hfx. volcanii* indicates that most of the core biosynthetic pathway for the synthesis of spermidine is present (Figure 1(a)). Two forms of ADC are found in Archaea [37]. The first type is a pyruvoyl-dependent enzyme, and the corresponding gene (*TK0149*) is essential for growth of* T. kodakarensis* [65]. The second is a paralog of AdoMet decarboxylase (AdoMet DC) that can decarboxylate arginine, found mainly in Crenarchaeota [37]. Like* T. kodakarensis*,* Hfx. volcanii* harbors a homolog of the pyruvoyl-dependent type encoded by* HVO_1958* (Figure 1(a)) [66].

The next step in the proposed archaeal spermidine pathway is the formation of putrescine by hydrolysis by the agmatinase/agmatine ureohydrolase (AUH) enzyme. The AUH enzymes from* Pyrococcus horikoshii* and* Methanocaldococcus jannaschii* have been biochemically characterized [67, 68], but no archaeal mutant was ever constructed. An agmatinase family member,* HVO_2299*, is found in* Hfx. volcanii* and could possibly catalyze this reaction (Figure 1(a)) [66].

Finally, spermidine synthase transfers the 3-aminopropyl moiety from decarboxylated adenosylmethionine (dcAdoMet) to putrescine. Two members of the spermidine synthase family are found in* Hfx. volcanii*:* HVO_0255* and* HVO_B0357* (Figure 1(a)) [66]. Deletion of the* HVO_B0357* gene did not give rise to any phenotypes under the conditions tested [44]. Interestingly, the N-terminal regions of* HVO_0255* and* HVO_B0357* and of halophilic homologs contain seven transmembrane domains (N-terminal transporter like domain) (Fig. S1A). The presence of the N-terminal transporter like domain in both of these proteins and the fact that the putrescine binding residues are not conserved in theses halophilic SpeE homologs (Fig. S1B) suggest that these proteins might have other as yet undetermined roles.

Whereas AdoMet DC is present in* T. kodakarensis* [69], no homologs of AdoMet DC were found in either* Hfx. volcanii* or more generally any sequenced halophiles (data not shown). This raises the question of the potential source of dcAdoMet in a putative spermidine synthase reaction.

Other genes putatively related to polyamine metabolism in* Hfx. volcanii* include the* HVO_0339* gene encoding the essential agmatidine synthase TiaS (Figure 1(a)) [44] and the* HVO_B0045* and* HVO_B0046* genes predicted to encode L-2,4-diaminobutyrate decarboxylase and L-2,4-aminobutyrate aminotransferase. It has been proposed that these two enzymes participate in the synthesis of a 1,3-diaminopropane-based siderophore such as rhizobactin 1021 or schizokinen [70].

**Figure 2:**
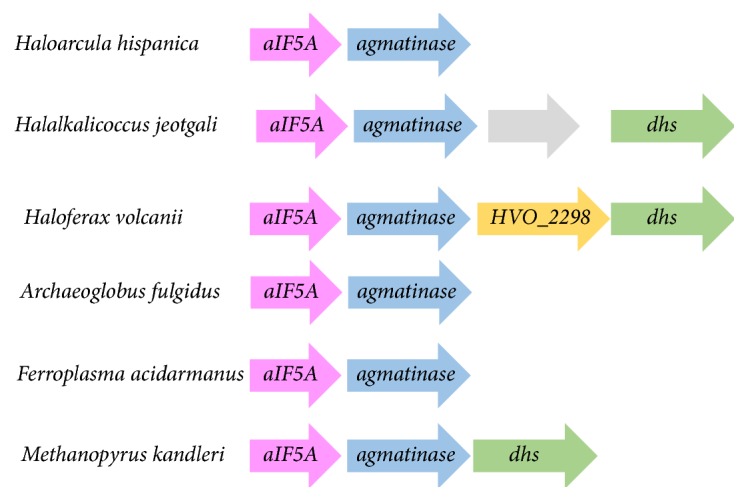
*HVO_2299* of* Hfx. volcanii* clusters with* aIF5A*. Physical gene clustering links genes encoding agmatinase, DHS, and aIF5A in Euryarchaeota. In pink,* aIF5A *(*HVO_2300* in* Hfx. volcanii*); in blue, agmatinase (*HVO_2299* in* Hfx. volcanii); *in yellow, gene encoding* HVO_2298*; DHS (*HVO_2297* in* Hfx. volcanii*). Arrows represent the genes. Physical clustering was analyzed on the STRING database.

Two members of the DHS family are found in* Hfx. volcanii HVO_2297* and* HVO_B0182 *[66]. The* HVO_2297* gene is physically clustered with the gene encoding aIF5a (*HVO_2300*) (Figure 2). To our knowledge, the activity of* HVO_2300* (as aIF5A) has never been experimentally validated, the gene is essential [12]. Hence,* HVO_2297* is a potential candidate for the canonical DHS enzyme and* HVO_B0182* could be involved in homospermidine synthesis as proposed by [37] (Figure 1(a)).

As summarized in Figure 1, metabolic reconstruction gives only a tentative picture of polyamine metabolism (Figure 1(a)) and the aIF5A modification pathway (Figures 1(b), 1(c), and 1(d)) in* Hfx. volcanii*. DHS could transfer the aminobutyl group from spermidine to aIF5A (Figure 1(b)) but it is not clear if the spermidine pathway is present in this organism since no AdoMet DC gene could be identified (Figure 1(a)). Other possibilities already partially discussed by [37] are that the* Hfx. volcanii *DHS (i) transfers directly putrescine (Figure 1(b)) or the aminobutyl group of agmatine to aIF5A (Figure 1(c)) and (ii) transfers agmatine to aIF5A, with the agmatinase acting on the modified protein substrate to produce the deoxyhypusinylated derivative (Figure 1(d)).

### 3.2. *Hfx. volcanii* aIF5A Is Expressed Throughout the Growth Curve and Deoxyhypusinylated* In Vivo*

**Figure 3:**
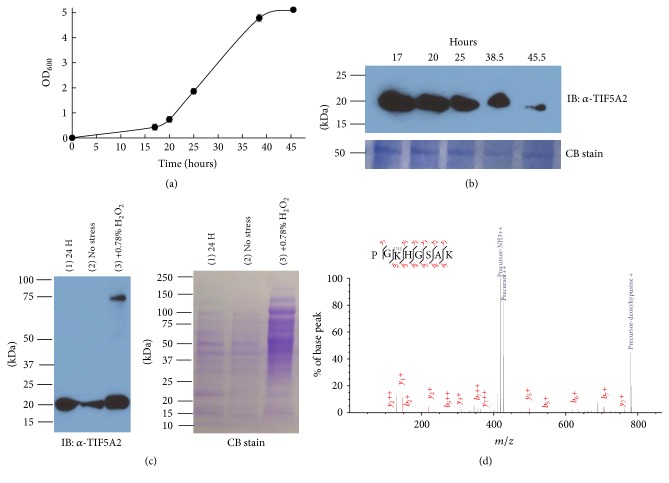
aIF5A produced in* Hfx. volcanii* is deoxyhypusinylated and sensitive to oxidative stress. (a)* Hfx. volcanii* H26 was grown at 42°C in ATCC974 (shaking at 200 rpm). 2 mL of samples at OD_600_ = 0.043 was harvested along the growth curve. (b) H26 cells pellets (2 mL at OD_600_ = 0.043 of the culture) were resuspended in SDS-PAGE loading buffer and boiled for 15 min. Equivalent protein loading was based on OD_600_ of cell culture (0.086 OD_600_ units per lane), as demonstrated by staining with Coomassie Brilliant Blue R-250 (CB stain, with representative 55 kDa protein band of* Hfx. volcanii* presented). Proteins were separated via 4–15% reducing SDS-PAGE. aIF5A was detected by *α*-aIF5A (anti-TIF5A2) immunoblot (IB). The experiments were performed with three biological replicates and the detection by anti-TIF5A2 or anti-TIF5A3. The molecular mass indicated is in kDa. (c) Effect of oxidative stress on aIF5A level. The cells were grown for 24 hours and then treated or not treated with 0.78% H_2_O_2_. Equivalent protein loading was based on OD_600_ of cell culture (0.086 OD_600_ units per lane). Lane 1, cells collected at 24 hours; lane 2, cells collected at +4 hours after no stress (control experiment); lane 3, cells collected at +4 hours after 0.78% H_2_O_2_ treatment. Proteins were separated by 4–15% reducing SDS-PAGE. aIF5A was detected via *α*-aIF5A (anti-TIF5A2) immunoblot (IB). The molecular mass indicated is in kDa. (d) Identification of the deoxyhypusine modification by LC-MS/MS analysis. The purified aIF5A His-C-term was loaded on a SDS-PAGE 12%. The proteins were detected by staining with Coomassie Blue (Fig S2B), and the protein band was cut and analyzed by LC-MS/MS analysis. Fragmentation spectrum for deoxyhypusinylated peptide PGKHGSAK is shown; xiSPEC (http://spectrumviewer.org/) was used for annotation.

The* Hfx. volcanii* gene predicted to encode aIF5a (*HVO_2300*) is essential [12], but little else is known about this gene/protein in this organism. To obtain a better understanding of aIF5A in Archaea and specifically in halophiles, aIF5A levels at different growth phases were monitored by Western blot using* Hfx. volcanii* anti-aIF5A antibodies (TIF5A2 and TIF5A3) (Figures 3(a) and 3(b)). Both antibodies independently detected aIF5A at around 20 kDa for an expected theoretical mass of 14.2 kDa. The observed size difference is most likely due to the known slow mobility of halophilic proteins on SDS-PAGE [71–73]. Similar levels of the aIF5A protein were detected for up to 25 hours of growth, after which the levels of aIF5A diminished (Figure 3(b)). Few studies have the regulation of eIF5A in Eukaryotes but it has been suggested that eIF5A levels are regulated in a proteasome-dependent manner [74, 75]. The effects of different stresses (proteasome inhibitor, cold shock, and oxidative stress) on the level of aIF5A were therefore monitored (as described in Section 2). We did not notice any effect of the proteasome inhibitor (Fig. S2A) or cold shock on the level of aIF5A (data not shown). Interestingly, the presence of 0.78% (w/v) H_2_O_2_ led to a change in the cell lysate protein pattern (Figure 3(c)). For the same quantities of cells (measured by OD), an additional higher molecular weight band (around 85 kDa) was observed (Figure 3(c)). The band at 85 kDa could be the result of protein modifications or aggregation due to the stress. Additional work is needed to understand the mechanisms that regulate aIF5A in Archaea and more generally the effects of oxidative stress on the* Hfx. volcanii* proteome.

In order to determine whether the* Hfx. volcanii* aIF5A is modified by hypusine or deoxyhypusine, the protein was purified to an apparent homogeneity as an 18 kDa protein (Fig. S2B) from strain VDC2577 (TIF5A-C-term His integrant)* via* affinity chromatography. We were able to detect the deoxyhypusine/hypusine modification of the purified aIF5A using the IU-88 antibody [55] (kind gift of Dr. Mirmira) (Fig. S2B). The IU-88 antibody failed to detect the modified aIF5A on cell lysates throughout the different growth phases (data not shown). As this antibody recognizes both the deoxyhypusine and hypusine forms of eIF5A [55], the 18 kDa protein band was cut out from the SDS-PAGE, digested with LysC, and analyzed by mass spectrometry (Figure 3(d)). This analysis confirmed that the purified protein is aIF5A-modified with deoxyhypusine at the conserved lysine (position 36) (Figure 3(d)). Furthermore, when comparing the intensities for unmodified, hypusinylated, and deoxyhypusinylated PGKHGSAK peptides, approximately 99% of the aIF5A protein is deoxyhypusinylated. Our results confirm the previous observation by amino acid analysis of the presence of deoxyhypusine in halophiles [32] and show for the first time by MS that* Hfx. volcanii* aIF5A is deoxyhypusinylated. Our data suggest that* Hfx. volcanii *aIF5A is exclusively deoxyhypusinylated in the cell, as no pool of unmodified aIF5A was detected in the purified fraction and correlates with the absence of DOOH homologs in this organism. Indeed, in Eukaryotes hypusination of eIF5A appears to be constitutive as hypusination occurs shortly after protein synthesis with no evidence of modification turn-over [30, 31, 76, 77]. We propose that the deoxyhypusinylated aIF5A is the active form in* Hfx. volcanii*, as it has been shown in* S. cerevisiae* that the partially modified deoxyhypusine eIF5A was functional [1, 3].

### 3.3. Intracellular Polyamines Composition of* Hfx. volcanii*

**Figure 4:**
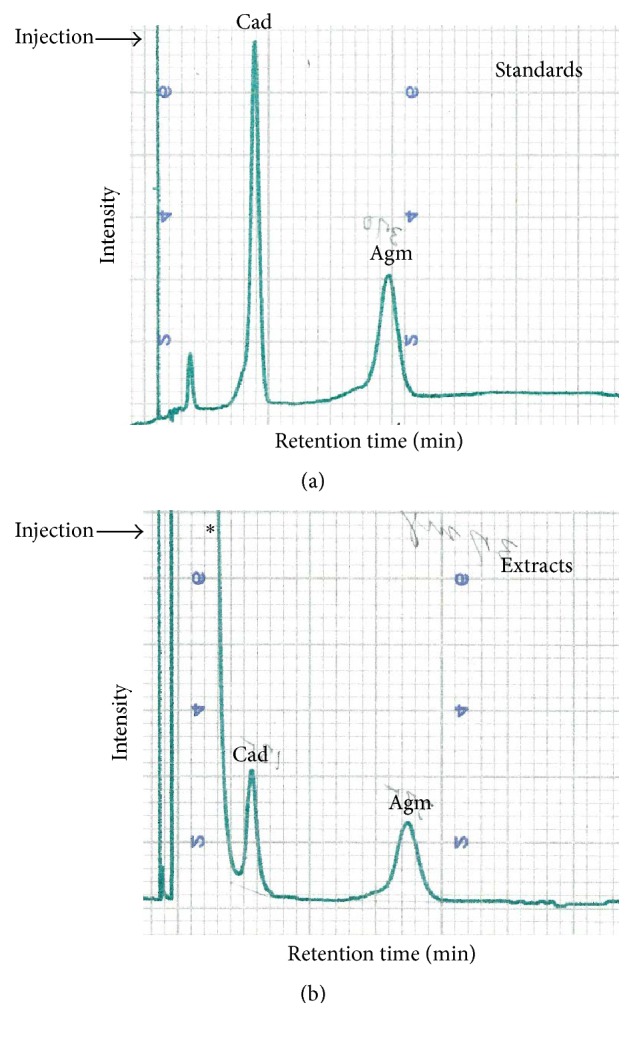
Intracellular polyamines analyses in* Hfx. volcanii* H26. H26 was grown at 42°C in Hv_min medium. Intracellular polyamines from* Hfx. volcanii* H26 were extracted after 20 hours of growth. (a), peak standards; (b), samples after 20 hours of growth. Thirty-seven mg of extracts was injected. The ratio agmatine/cadaverine is 6.96 ± 3.28. The injection is indicated by the arrow; ^*∗*^unexpected noise derived from buffer.

Previous polyamine composition analysis studies of halophilic Archaea were performed in various media types depending on the study and specific organism investigated, making it difficult to compare results. Indeed, when cells were grown in rich medium contamination with salvaged polyamines can occur [40] as summarized in Table S1. To obtain more information, the intracellular polyamine composition in* Hfx. volcanii* H26 was analyzed by HPLC. Intracellular polyamines were extracted at different growth stages (20 hours, 35 hours, and 40 hours) of cells grown in minimal medium (Hv_min). Two major peaks were detected at the position of agmatine and cadaverine (Figure 4). In these conditions, no changes in polyamines composition during growth were observed (Figure 4 and Fig. S3). Agmatine was found to be the more abundant polyamine at all growth stages (Figure 4(b) and Fig. S3). This result confirms previous studies assigning agmatine as the major polyamine in halophilic archaea and in* Hfx. volcanii* (Table S1) [41, 78–80]. Hamana et al. reported the presence of a small amount of putrescine when* Hfx. volcanii* was grown in rich medium [41] (Table S1) (medium NCIMB 2012 at 30°C). This result was not reproduced in minimal medium. The presence of cadaverine was not predicted from the initial metabolic reconstruction (Figure 1(a)). It has been shown in some Bacteria that the L-2,4-diaminobutyrate decarboxylase, involved in siderophore synthesis, can also function as a lysine decarboxylase [70]. Our results suggest that this could also be the case in* Hfx. volcanii* as the gene* HVO_B0045* is predicted to encode L-2,4-diaminobutyrate decarboxylase. Cadaverine had previously been reported in a few halophilic Archaea (Table S1) and it might have a role in resistance of these organisms to high salt since this metabolite has been linked to pH and salt resistance [81, 82]. The absence of spermidine is also consistent with previous work (Table S1) and with the fact that no dcSAM synthesis enzyme is encoded by* Hfx. volcanii *(Figure 1(a)). These results raise the question of the role of the SpeE homologs (*HVO_0255* and* HVO_B0357*) in this halophile.

### 3.4. Arginine Decarboxylase Is Essential for the Growth of* Hfx. volcanii*

**Figure 5:**
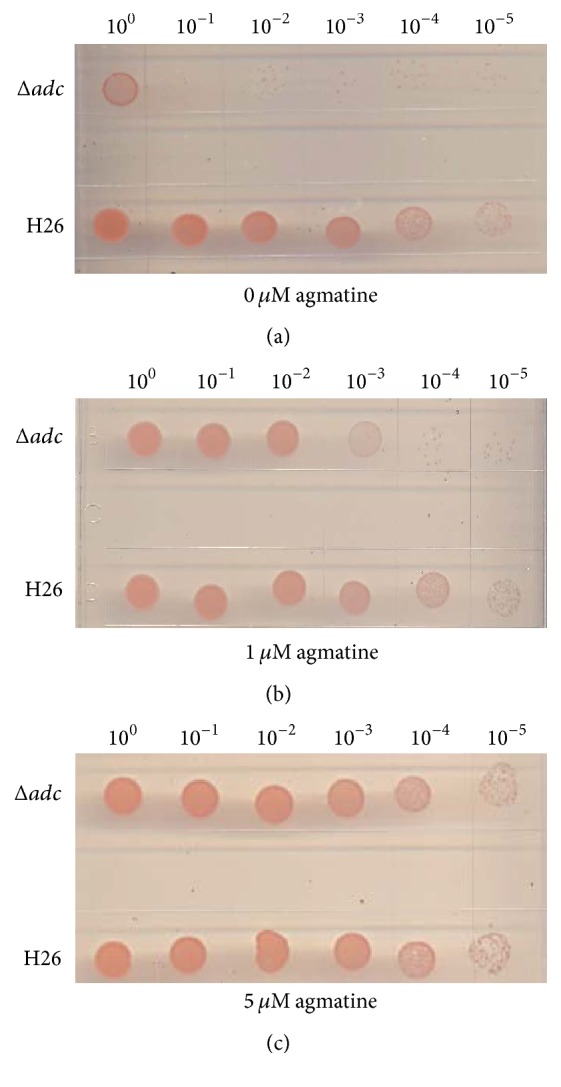
Agmatine is essential for the growth of the* Hfx. volcanii *Δ*adc* strain.* Hfx. volcanii* H26 (WT, parent) and Δ*adc (HVO_1958)* were diluted to an OD_600_ of 1 and then spot-plated (15 *μ*L) on solid agar Hv_min medium in serial dilutions as indicated: (a) in absence of agmatine; (b) in presence of 1 *μ*M agmatine; (c) in presence of 5 *μ*M agmatine. Each experiment was performed with two biological replicates and three technical replicates (for description of biological versus technical replicates).

To confirm that* HVO_1958* encodes an arginine decarboxylase, the gene was deleted (Fig. S4A-B) and the growth of the deletion strain was compared to its parent (H26) at 42°C in the absence (Figure 5(a)) or presence of agmatine (Figures 5(b) and 5(c)). The Δ*HVO_1958 strain* is auxotrophic for agmatine and requires 5 *μ*M agmatine for full growth (Figure 5(c)) and was therefore renamed Δ*adc*. Partial growth was observed at lower agmatine concentrations (Figure 5(b)) but no growth was observed for <1 *μ*M agmatine (data not shown). As already seen with the* T. kodakarensis *Δ*adc* strain [65], growth of the* Hfx. volcanii *Δ*adc* mutant could only be rescued by agmatine and not by any of the other tested polyamines such as putrescine, spermidine, cadaverine, or ornithine (Fig. S5). Moreover, the addition of polyamines in the media had no effect on the growth of the parent strain* Hfx. volcanii* H26 (Fig. S5). These genetic experiments support the model in which the* HVO_1958* encoded ADC is the first enzyme of polyamine synthesis in* Hfx. volcanii* producing the essential intermediate agmatine (Figure 1(a)). Our genetic studies corroborate our polyamines analyses since deleting the predicted arginine decarboxylase gene (*HVO_1958) *led to an agmatine auxotroph strain. The agmatine produced by* Hfx. volcanii* ADC is certainly a precursor to the formation of the essential agmatidine tRNA modification catalyzed by TiaS (Figure 1(a)), but it could also be required for the formation of the essential deoxyhypusine modification as a direct substrate of DHS (Figures 1(c) and 1(d)) or as the precursor of the final polyamine substrate for the DHS reaction (Figures 1(a) and 1(b)).

### 3.5. *HVO_2299*, Gene Encoding an Agmatinase-Like Protein, Is Essential

The metabolic reconstruction described above suggests a role of* HVO_2299* as an agmatinase involved in forming putrescine from agmatine (Figure 1(a)). However, neither putrescine nor the downstream product spermidine was detected in our polyamine analysis of* Hfx. volcanii *(Figure 4). To gain further insight into the role of agmatinase, the gene neighborhood of the* HVO_2299* was explored (Figure 2). Our analysis uncovered strong physical clustering associations between the aIF5A and agmatinase encoding genes, with 39% of the archaeal genomes present in the STRING database (out of the 130 archaeal genomes) showing this association (Figure 2). These clusters were almost exclusively observed in Euryarchaeota and were present in ~62% of the Euryarchaeota and in the unclassified archaea halophilic archaeon DL31. Interestingly, the association* aIF5A*/*agmatinase* was not found in the Crenarchaeota phylum, where* aIF5A* was found associated with genes encoding DNA topoisomerase IV subunit B, DNA topoisomerase VI subunit A, and DHS (data not shown). Physical clustering evidence strongly pointed to a role of HVO_2299 in aIF5a modification. This agmatinase-like protein could be involved in the formation of the intermediate putrescine (Figure 1(a)), even if it has not been detected in our analyses or could directly hydrolyze a modified aIF5a precursor to form the final deoxyhypusine modification (Figure 1(d)).

To investigate if* HVO_2299* is essential for* Hfx. volcanii* cell viability, we attempted to delete the gene from its chromosomal locus. Around 220 candidate colonies were screened but no deletion mutant was obtained suggesting the essentiality of this gene. The deletion of the gene failed even in the presence of 1 mM putrescine. We then proceeded to clone the* HVO_2299* into pPT002 plasmid, placing the gene under the tryptophanase promoter [49, 83]. The plasmid was transformed into the “pop-in strain.” In this background, it was possible to delete the chromosomal copy of the* HVO_2299 *(Fig. S4 C-D), again strongly suggesting essentiality of the gene. However, growth was still observed in the absence of tryptophan, certainly caused by the known the leakiness of the P_TNA_ promoter in multicopy plasmids [49].

The fact that the* HVO_2299 *gene encoding an agmatinase-like protein is essential even in the presence of putrescine, as well as the absence of any detected intracellular putrescine, suggests that this enzyme is not involved in the formation of putrescine from agmatine in* Hfx. volcanii*. One, however, cannot rule out the possibility that putrescine is not imported in this organism and has to be produced* de novo* and immediately used to modify aIF5a by DHS (Figure 1(b)), reducing its accumulation to below detectable limits. This is unlikely as all the genes encoding spermidine/putrescine ABC transporter [PotA1 (*HVO_A293*) and PotA2 (*HVO_A293*), PotB (*HVO_A300*), PotC (*HVO_A297*) and PotD* (HVO_A299)*] are found in the* Hfx. volcanii* genome on the pHV4 plasmid in a physical cluster (data not shown). If putrescine is transported in the cell, then the essentiality of* HVO_2299* would favor a model where the agmatinase protein might be hydrolyzing the agmatine moiety after its transfer to aIF5A (Figure 1(d)). Further efforts are required to undercover the function of the agmatinase-like protein in* Hfx. volcanii*.

### 3.6. Deoxyhypusine Synthase Activities in Euryarchaeota

**Figure 6:**
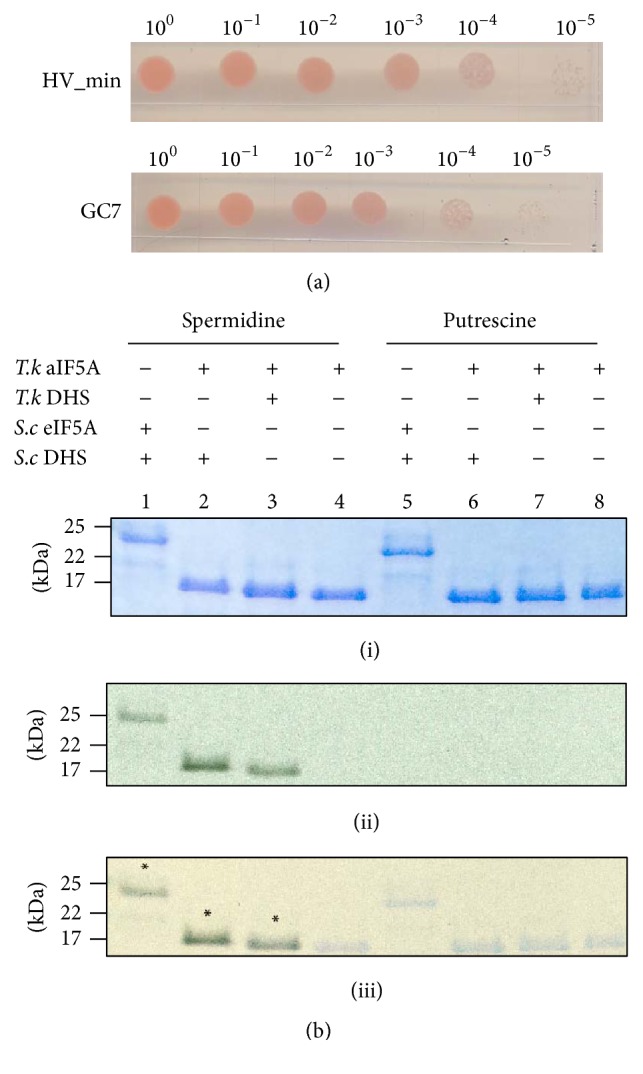
*Hfx. volcanii* is insensitive to GC7 and spermidine is the substrate of* T. kodakarensis *DHS. (a) Effect of GC7 on the growth of H26.* Hfx. volcanii* H26 (WT, parent) was diluted to OD_600_ of 1 and then spot-plated (15 *μ*L) on solid agar Hv_min medium in serial dilutions as indicated in absence or in presence of 1 mM GC7. Each experiment was performed with two biological replicates and three technical replicates. (b) Detection of* T. kodakarensis* aIF5A (lanes 2, 3, 4, 6, 7, and 8) and* S. cerevisiae* eIF5A (lanes 1 and 5) modification using [^14^C] spermidine or [^3^H] putrescine as substrates in presence of* T. kodakarensis *DHS (lanes 3 and 7) or* S. cerevisiae* DHS (lanes 1, 2, and 5). The* in vitro* assay was resolved on a 16.5% tricine polyacrylamide gel. The dried gels were exposed to autoradiography films to visualize possible modifications. The assembly of each reaction is depicted above. (i), Coomassie Blue staining; (ii), exposed autoradiography film; (iii), overlay of the Coomassie Blue stained gel and the exposed film; *∗* indicates the presence of the radioactivity; molecular mass markers are indicated in kDa;* T.k*,* Thermococcus kodakarensis*;* S.c*,* Saccharomyces cerevisiae*.

*N* ^1^-Guanyl-1,7-diaminoheptane (GC7) is a very efficient inhibitor of DHS in eukaryotes. This homolog of spermidine inhibits the first step in the hypusination pathway by binding to DHS [28, 29]. Cultures of* Sulfolobus acidocaldarius* DSM 639,* Sulfolobus solfataricus* DSM,* Halobacterium halobium* DSM 670, and* Haloferax mediterranei* DSM were shown to be sensitive to GC7 [33]. To determine the effect of GC7 on* Hfx. volcanii* culture, H26 was grown in the absence or presence of 1 mM GC7 at 42°C on Hv_min agar. As shown in Figure 6(a), we did not observe any effect of GC7 on the growth of H26 even if it should be transported by the predicted spermidine/putrescine ABC transporter present in* Hfx. volcanii*. The same results were observed in liquid cultures, although the compound did affect growth of* Saccharomyces cerevisiae* cultures as expected (data not shown).

To determine whether the DHS of* Hfx. volcanii* transfers the 4-aminobutyl moiety from spermidine to the* Hfx. volcanii* aIF5A lysine side chain, the protein was expressed from* Hfx. volcanii *(LSP5021). The protein T7-His-DHS was purified as a tetramer with an estimated molecular weight of around 148 kDa (Fig S6). The oligomerization state of the purified DHS is in agreement with the crystal structure of the Human and other characterized DHS enzymes [84–86]. The substrate of the reaction,* Hfx. volcanii* aIF5A-C-term-His, was overexpressed in* E. coli* and purified in two steps. The purified* Hfx. volcanii* T7-His-DHS did not show any activity under varying aIF5A concentration and substrate concentration (spermidine, agmatine, cadaverine, and putrescine) (data not shown). In an effort to determine if the lack of activity of the DHS was due to the presence of the T7-His tag, the protein was expressed without tag and the DHS activities were tested in the* Hfx. volcanii* cell lysate. No DHS activity was detected in the cell lysate (Fig. S7). The lack of activity was not due to a defect of the* Hfx. volcanii* aIF5A-C-term-His substrate, since the* S. cerevisiae* DHS catalyzed the transfer of 4-aminobutyl moiety from spermidine to the* Hfx. volcanii* aIF5a-C-term-His (Fig. S7, lane 11). DHS sequences from Archaea that are sensitive to GC7 [34] (Fig. S8, group A) and halophilic Archaea that harbor the polyamine cadaverine like* Hfx. volcanii* (Fig. S8, group B) were aligned and the active site and spermidine substrate binding residues mapped. If the active site K329 (Human, P49366) is strictly conserved from Human to all archaeal DHS proteins, the spermidine binding sites residues are not (Fig. S8). One of the spermidine binding residues (H288) is strictly conserved in Eukarya and in the group A Archaea (sensitive to GC7) but this residue is not conserved in the group B Archaea (that harbor cadaverine). Those differences could explain why GC7 and spermidine do not bind* Hfx. volcanii* DHS. This study opens the question of DHS in halophile organisms.

As a further control, the* in vitro* DHS activity of another member of the Euryarchaeota was explored. Recombinant DHS from the thermophile* T. kodakarensis* and its substrate aIF5A were purified and the activity was tested in presence of radioactive spermidine or putrescine. Similar to the eukaryotic enzyme (Figure 6(b), lane 1), the DHS of* T. kodakarensis* transfers 4-aminobutyl from spermidine to* T. kodakarensis* aIF5A* in vitro* (Figure 6(b), lane 3). No transfer was observed in absence of the enzyme (Figure 6(b), lane 4) or in presence of putrescine (Figure 6(b), lane 7). The modified* T. kodakarensis* aIF5A was analyzed by mass spectrometry demonstrating the presence of the deoxyhypusine on the conserved lysine K42 (Fig. S9).

The absence of any effect of the GC7 on* Hfx. volcanii* growth as well as the inability to observe transfer of 4-aminobutyl suggests that spermidine is not the substrate of the* Hfx. volcanii* DHS, corroborating the metabolic reconstruction and the fact that no intracellular spermidine was detected. We cannot rule out the possibility that GC7 was not imported into the cells, despite the presence of the spermidine/putrescine ABC transporter genes and that the* Hfx. volcanii* DHS was purified in an inactive form. Unfortunately, our attempts to test the different models (Figures 1(b), 1(c), and 1(d)) by testing the activity of the* Hfx. volcanii* DHS protein* in vitro* remained unsuccessful. We were able to purify an active aIF5A that could be deoxyhypusinylated with the* S. cerevisiae* DHS using spermidine as aminobutyl donor, but all attempts with* Hfx. volcanii *DHS failed using spermidine, putrescine, or agmatine as donors. Nevertheless, we could show for the first time the* in vitro* activity of the* T. kodakarensis* DHS with the transfer of 4-aminobutyl from spermidine to aIF5A, as in Eukaryotes.

## 4. Conclusion

We have demonstrated that* Hfx. volcanii* aIF5A is deoxyhypusinylated. The absence of spermidine in* Hfx. volcanii* was confirmed, and the major polyamines in this halophile were found to be agmatine and cadaverine. We propose that deoxyhypusine synthesis in* Hfx. volcanii* differs from the canonical eukaryotic pathway. Based on our observation that (i) GC7, the spermidine analog that inhibits yeast DHS, does not inhibit* Hfx. volcanii* growth, (ii) the* agmatinase-like* gene that clusters with the* dhs *gene is essential for the growth of* Hfx. volcanii* even in the presence of putrescine, and (iii) there is no activity of the* Hfx. volcanii* DHS with spermidine, we favor the model in Figure 1(d), where DHS transfers agmatine to the aIF5A lysine and the agmatinase enzyme is required to produce deoxyhypusine.

## Supplementary Material

Table S1 of the supplemental information summarizes cellular polyamines composition in Archaea. The plasmids, strains and oligonucleotides of this study are listed in table S2 and S3. The supplemental information contains additional experiments listed from Fig. S1 to Fig. S9.

## Nonstandard Abbreviations

ADC: Arginine decarboxylase
AdoMet DC: AdoMet decarboxylase
Amp: Ampicillin
AUH: Agmatinase/agmatine ureohydrolase
dcAdoMet: Decarboxylated adenosylmethionine
DHS: Deoxyhypusine synthase
DOHH: Deoxyhypusine hydroxylase
eIF5A: Eukaryotic initiation factor 5A
aIF5A: Archaeal initiation factor 5A
GC7: *N* ^1^-Guanyl-diaminoheptane
HTP: Hydroxyapatite column
LC: Liquid chromatography
TCA: Trichloroacetic acid
IF5a: Translation initiation factor 5A.
