# Supplementary material for: Deciphering the Translation Initiation Factor 5A Modification Pathway in Halophilic Archaea
Source: Archaea. 2016 Dec 8;2016:7316725. doi: 10.1155/2016/7316725 (PMC5178350; doi:10.1155/2016/7316725)
Supplement: Supplementary file 1 — Table S1 of the supplemental information summarizes cellular polyamines composition in Archaea. The plasmids, strains and oligonucleotides of this study are listed in table S2 and S3. The supplemental information contains additional experiments listed from Fig. S1 to Fig. S9. [file 7316725.f1.pdf]

Supporting information: Deciphering the Translation Initiation Factor 5A Modification Pathway in *Halophilic Archaea*

Authors: Laurence Prunetti[1], Michael Graf [2], Ian K. Blaby[1#], Lauri Peil[3], Andrea M Makkay[4], Agata L Starosta[2%], R Thane Papke[4], Tairo Oshima[5], Daniel N Wilson[2] and Valérie de Crécy-Lagard[1]*

[1]Department of Microbiology and Cell Science, Institute for Food and Agricultural Sciences and Genetic Institute, University of Florida, P.O. Box 110700, Gainesville, FL 32611-0700, USA.

[2] Gene Center and Department for Biochemistry, University of Munich, 81377 Munich, Germany; Center for Integrated Protein Science Munich, University of Munich, 81377 Munich, Germany

[3] Faculty of Science and Technology, Institute of Technology, University of Tartu, Tartu, Estonia

[4]Department of Molecular and Cell Biology, University of Connecticut, 91 N. Eagleville Rd, Storrs, CT, 06269, USA.

[5] Institute of Environmental Microbiology, Kyowa Kako Co. Ltd., Tadao 2-15-5, Machida 194-0035, Japan

[#] Current address: Biology Department, Brookhaven National Laboratory, 50 Bell Avenue, Building 463, Upton, NY 11973, USA

%Current address: Centre for Bacterial Cell Biology, Institute for Cell and Molecular Biosciences, University of Newcastle, Newcastle upon Tyne, United Kingdom

[*]To whom correspondence should be addressed.

	Agm	Spd	Spm	Put	Cad	Hspd	Ref
**Euryarchaeota**							
Halorubrum lacusprofondi JCM 8891#	2.20	-	-	1.20	-	-	[1]
Halorubrum saccharovorum JCM 8865#	4.80	-	-	0.65	-	-	[1]
Halobacterium salinarium JCM 8978#	1	-	-	0.20	-	-	[1]
Halorubrum sodomense JCM 8880#	2.60	-	-	-	-	-	[1]
Halorubrum trapanicum JCM 8979#	1.50	-	-	0.10	-	-	[1]
Halobacterium salinarum NCIMB 786#	6.10	-	-	-	-	-	[1]
Halobacterium salinarum ATCC 43214#	8	-	-	0.10	-	-	[1]
Haloarcula hispanica JCM 8911#	3.80	-	-	0.10	-	-	[1]
Haloarcula aidinensis JCM 10024#	1.60	-	-	-	0.10	-	[2]
Haloarcula japonica JCM 7785#	1.20	-	-	-	-	-	[1]
Haloarcula marismortui JCM 8966#	4.50	-	-	-	-	-	[1]
Haloarcula quadrata JCM 11048#	0.70	-	-	-	0.05	-	[2]
Haloarcula vallismorits JCM 8877#	1.50	-	-	-	-	-	[1]
Haloferax denitrificans ATCC 35960#	2.20	-	-	-	-	-	[1]
Haloferax gibbonsii ATCC 33959#	1	-	-	-	-	-	[1]
Haloferax mediterranei JCM 8866#	2	-	-	-	-	-	[1]
Haloferax volcanii NCIMB 2012#	4.50	-	-	0.10	-	-	[1]
Halococcus morrhuae ATCC 17082#	2.10	-	-	-	-	-	[1]
Halococcus saccharolyticus ATCC 49257#	4.50	-	-	-	-	-	[1]
Haloterrigena thermotolerans	1.90	-	-	-	0.07	-	[2]
Natronobacterium gregoryi NCIMB 2189#	0.40	0.70	0.10	0.4	-	-	[1]
Natronomonas pharaonis JCM 8858#	0.60	-	-	0.10	-	-	[1]
Halorubrum vacuolatum JCM 9060#	1.20	5.30	0.30	0.10	-	-	[1]
Natronococcus occultus JCM 8859#	0.40	-	-	0.20	-	-	[1]
Natrialba magadii NCIMB 2190#	0.30	0.60	0.10	0.50	-	-	[1]
Ferroplasma acidophilum JCM 10970$	0.17	1.50	-	-	-	-	[3]

Thermoplasma acidiphilium JCM 9062[$]	0.03	1	-	-	-	-	[3]
Thermoplasma volcanium JCM 9571[$]	0.13	1.2	-	-	-	-	[3]
Thermococcus zilligii JCM10554[$]	0.15	0.82	-	-	-	-	[3]
Thermococcus waiotapuensis JCM10985[$]	0.10	1.24	0.02	0.04	-	-	[3]
Thermococcus aegaeus JCM10828[$]	0.10	1.40	0.10	0.26	-	-	[3]
Pyrococcus glycovorans AL585[$]	0.06	0.80	-	-	-	-	[3]
Pyrococcus furiosus JCM8422[$]	-	0.06	0.15	0.06	-	-	[3]
Pyrococcus horikoshii JCM9974[$]	0.60	0.90	-	0.05	-	-	[3]
Pyrococcus woesei JCM8421[$]	-	0.28	-	0.16	-	-	[3]
Methanococcus vannielii[$]	0.16	0.76	0.07	0.07	-	-	[4]
Methanococcus vannielii[%]	-	28.5	-	3.40	-	-	[5]
Methanocaldococcus jannaschii[$]	1.50	0.60	1.50-	0.04	-	-	[2]
Methanosarcina mazei S-6[%]	-	-	-	19.20	-	6.90	[5]
Methanosarcina barkeri MS[%]	-	-	-	18.70	-	4.10	5]
**Crenarchaeota**							
Sulfolobus tokodaii JCM10545[$]	-	1.86	0.05	0.05		0.01	[3]
Sulfolobus solfataricus JCM 11322[$]	-	1.40	-	0.10	-	0.04	[3]
Metallosphaera sedula JCM 9064[$]	-	1.68	-	0.55	-	0.04	[3]
Acidilobus aceticus JCM 11320[$]	-	1.51	-	0.10	-	-	[3]
Thermodiscus maritimus JCM 11597[$]	-	1.15	-	0.32	0.12	-	[3]
Pyrobaculum arsenaticum[$]	-	1.40	-	-	-	-	[3]
Pyrobaculum oguniense JCM 10595[$]	-	0.85	0.55	-	-	-	[3]
Pyrobaculum aerophilium JCM 9630[$]	-	0.24	0.10	-	-	-	[3]
Pyrobaculum islandicum JCM 9189[$]	-	0.85	0.50	-	-	-	[3]
Pryrobaculum organotrophum JCM9190[$]	-	1.3	0.40	0.20	-	-	[3]
Vulcanisaeta distributa JCM 11212[$]	-	0.55	0.02	-	0.10	-	[3]
Vulcanisaeta souniana JCM 11219[$]	-	1.80	0.02	-	-	-	[3]
Sulfolobus acidocaldarius[$]	-	1.40	-	0.10	-	0.04	[3]

Table S1: Examples of cellular polyamines detected in archaea. The table is a non-exhaustive list of polyamines detected in some Archaea. Just the presence of the polyamines agmatine, putrescine, spermidine, spermine, cadaverine and homospermidine were reported in the table. -, non-detected; #, polyamines concentration in nmoles/g wet cell; $, polyamines concentration in μmoles/g wet cell. %, polyamines concentrations μmol/ g dry cells; Agm, agmatine; Spd, spermidine; Spm, spermine; Put, putrescine; Cad, cadaverine; Hspd, homospermidine; Ref, reference; JCM, Japan Collection of Microorganism; NCIMB, National Collections of Industrial, Marine and Food Bacteria; ATCC, American Type Culture Collection.

Strain, plasmid	Phenotype, genotype and/or description[a]	Ref/Source
E. coli		
TOP10	F⁻ *mcrA* Δ(*mrr-hsdRMS-mcrBC*) Φ80*lac*ZΔM15 Δ*lacX74 recA1 araD139* Δ(*ara leu*)*7697 galU galK rpsL* (Str[r]) *endA1 nupG*	Invitrogen
Inv110	F´ {*tra*Δ36 *proAB lacIq lacZ*Δ*M15*} *rpsL* (*Str[r]*) *thr leu endA thi-1 lacY galK galT ara tonA tsx dam dcm supE44* Δ(l*acproAB*) Δ(*mcrC-mrr*)102::Tn10 (Tet[r])	Invitrogen
BL21 (DE3)	*fhuA2 [lon] ompT gal (λ DE3) [dcm]* Δ*hsdS λ DE3 = λ sBamHIo* Δ*EcoRI-B int::(lacI::PlacUV5::T7 gene1) i21* Δ*nin5*	Novagen
Rosetta gammi 2 (DE3)	*Δ(ara-leu)7697 ΔlacX74 ΔphoA PvuII phoR araD139 ahpC galE galK rpsL (DE3) F'[lac+ lacIq pro] gor522::Tn10 trxB pRARE2 (CamR, StrR, TetR)*	Novagen
Hfx. volcanii		
H26	DS70 Δ*pyrE2*	[6]
VDC3253	H26 Δ *HVO_1958*	This work
LSP5061	H26 Δ *HVO_2299* transformed with pLSP21	This work
VDC2577	H26 *TIF5A-C-term His integrant*	This work
LSP5047	H26 transformed with pLSP20	This work
LSP5021	H26 transformed with pLSP23	This work

**Plasmids**		
pTA131	Amp^r; ColE1	[6]
pJAM202	Shuttle vector Amp^r; Nv^r	[7]
pIKB298	Amp^r; ColE1; *HVO_2299* deletion mutant construction	This work
pIKB313	Amp^r; ColE1; *HVO_1958* deletion mutant construction	This work
pIKB473	Amp^r; ColE1; Tif5a-His-C-term integrant construction	This work
pPT002	Amp^r; Nv^r; PJAM202 under ptna promoter	[8]
pLSP21	Amp^r; Nv^r; pPT002 carries *HV_22299*	This work
pLSP23	Amp^r; Nv^r; pJam202 carries *T7-His-DHS*	This work
pLSP24	Km^r; Pet28 carries *Hfx.volcanii aIFA5 C-term-His*	This work
pLSP20	Amp^r; Nv^r; pJAM202 carries *HVO_2297*	This work
pMG1	Km^r; pET28 carries *T. Kodakarensis aIFA5 N-term-His*	This work
pMG2	Km^r; pET8 carries *S. cerevisiae eIFA5-N-term-His*	This work
pMG3	Km^r; pET28 carries *T. kodakarensis dhs-N-term-His*	This work
pMG4	Km^r; pET28 carries S. cerevisiae *dhs-N-term-His*	This work
pAS1	Amp^r; pET21b carries *E.coli SpeA-C-term-His*	This work

Table S2. List of strains and plasmids used in this study. [a]Abbreviations: Amp^r, ampicillin resistance; Nv^r, novobiocin resistance ; Km^r, kanamycin resistance; Str^r, streptomycin resistance; Tet^r, tetracycline; Cterm His-, C-terminal poly-His_6 tag fusion protein; T7-His-, N-terminal tandem T7 tag and poly-His_6 tagged protein. The gene encoding aIF5A (*HVO_2300*) was amplified by PCR using the primers FW NCO1 TIFA and RV TIF5A-Cterm hist (Table S3), and cloned between the *NCo*I and *Blp*I sites of pET28a (+), adding an C-terminal hexa-histidine tag to give the plasmid pLSP24. The gene encoding DHS (HVO_2297) was first subcloned into pET28a (+). Briefly, the plasmid was treated with *Nco*I and *Nde*I to remove the sequence encoding for N-terminal hexa-histidine tag. The extremities were filled with

klenow polymerase (NEB). The gene encoding DHS (*HVO_2297*) was amplified by PCR using the primers (Table S3) FW Hist HVO_2297 and RV t7 HVO2297, and cloned between the *BamH*I and *Blp*I sites of the modified pet28 (+) adding a T7-N-terminal hexa-histidine tag. The sequence of the T7-His-DHS was PCR amplified with the primers FW202 NDe1 T7HDHS and RV DHS Blp1 (table S3), and cloned between the *Nde*I and *Blp*I sites of pJAM202 to give the plasmid pLSP23. The gene encoding DHS (*HVO_2297*) was amplified by PCR using the primers FW Nde1 DHS and RV DHS Blp1 (table S3), and clone between *Nde*I and *Blp*I of pJAM202 to give the plasmid pLSP20. The gene encoding agmatinase-like (*HVO_2299*) was amplified by PCR using the primers HV 2299 FW and HV 2299 RV, and cloned between *Nde*I and *BamH*I sited of pPT002 to give the plasmid pLSP21. The sequence of *T. kodakarensis aIF5A* and *S. cerevisiae eIF55* were PCR amplified using the primers aIF5_Tkod_FWD, aIF5A_Tkod_REV and eIF5A_Scer_FWD, eIF5A_Scer_REV respectively (table S3), and cloned between the *Sac*I and *Xho*I sites of pET28a (+) adding a N-term poly-His$_6$ to give the plasmid pMG1 and pMG2. The sequence of *T. kodakarensis dhs* and *S. cerevisiae dhs* were PCR amplified using the primers Dhs_Tkod_FWD, Dhs_Tkod_REV and Dhs_Scer_FWD, Dhs_Scer_REV respectively (Table S3), and cloned between the *Eco*RI and *Not*I sites of pET28a (+) adding a N-term poly-His$_6$ to give the plasmid pMG3 and pMG4. The sequence of speA was PCR amplified using the primers FOR Eco_speA and RV Eco_speA and then was cloned into pET21b using Megawhop cloning [9] to generate pAS1.

Oligonucleotide	Sequence (5'-3')	Function
aIF5A_Tkod_FWD	gatcagagctcatgggagacaagactaaggttcag	Amplification of *T. Kodakarensis* aIF5A gene for subsequent cloning into pET28
aIF5A_Tkod_REV	cgtatctcgagtcactcgcccctgatcttctttatc	
Eif5a_HIS_NF	cgggcccccctcgagcgattctctttccgttccag	Amplification of regions surrounding *aIIF5* (*HVO_2300*) to generate pIKB473
Eif5a_HIS_NR	ttagtgatggtgatggtgatgcggtatcaggaagctgct gacgatctttcgctggcctt	
Eif5a_HIS_CF	agcagcttcctgataccgcatcaccatcaccatcactaa acgggggacacagagatgtt	
Eif5a_HIS_CR	cgggctgcaggaattccgctcagatagacggattgg	

eIF5A_Scer_FWD	gatcagagctcatgtctgacgaagaacacacctttg	Amplification of *S.cerevisa* eIF5A gene for subsequent cloning into pET28
eIF5A_Scer_REV	cgtatctcgagttaatcagatcttggagcttccttgaa	
agmat NF	cgggcccccctcgaggcctgcccgatgtgaatc	
agmat NR	gacgcgttcatatgcgacgtggtggaagtcaacg	Amplification of regions surrounding *HVO_2299*, to generate pIKB298
agmat CF	gcatatgaacgcgtcgccgagaaggacgtagtcag	
agmat CR	cgggctgcaggaattcgacgaccgccatcatgc	
argDC_NF	cgggcccccctcgagaagcggacggactcgaag	Amplification of regions surrounding *HVO_1958*, to generate pIKB313
argDC_NR	gacgcgttcatatgcacatgaacacgattcgcgt	
argDC_CF	gcatatgaacgcgtcctacaccaccgcggtcac	
argDC_CR	cgggctgcaggaattcgtcgatctcacgtctgcg	
Dhs_Scer_FWD	gatcagaattcccatggatgtccgatatcaacgaaaaactc	Amplification of *S. cerevisae dhs* gene for subsequent cloning into pET28
Dhs_Scer_REV	cgtatgcggccgcttaattcttaactttttgattggtttacc	
Dhs_Tkod_FWD	gatcagaattcccatggatgaccgagccgaaagatatcgtc	Amplification of *T. kodakarensis dhs* gene for subsequent cloning into pET28
Dhs_Tkod_REV	cgtatgcggccgctcagggggctttcatcaccttccac	
HV 2299 FW	cggcatcatatgttccccggagcaac	To insert *HVO_2299* into pPT002
HV 2299 RV	aataacggatccttacgaccgcccggcgg	
ext f	aagcggacgactcgaag	To check *HVO_1958* deletion
ext r	cgcagacgtgagatcgac	
FW -391	atggcgaaagagcagaagcaggtgcgcgag	
RV -391	cgaaccagcggctcgatgcggtcgaagt	To check *HVO_2299* deletion
FW NCO1 TIFA	caacatccatggatggcgaaagagcagaagcaggtg	To construct *aIF5A C-term His*
RV TIF5A-Cterm hist	aataacgctcagcttagtgatggtgatggtgatggacgatctttcgctggccttcg	
FW Hist HVO_2297	cggcatggatccagccatcaccaccatc	To construct *T7-His-DHS*
RV t7 HVO2297	aataacgctcagcttactcgattcgctcgcgcgccgcggcgac	
FW202 NDe1 T7HDHS	cggcatcatatggctagcatgactg	To insert *T7- His-DHS* into

RV DHS Blp1	aataacgctcagcttactcgattcgctcgcg	pJAM202
FW Nde1 DHS	cggcatcatatgagcgaccacgacgat	To insert *HVO_2297* into pJAM202
RV DHS Blp1	aataacgctcagcttactcgattcgctcgcg	
FOR Eco_speA	gtttaactttaagaaggagatatacatatgtctgacgacatgtctatg ggtttgcc	To insert *speA* into pET21
REV Eco_speA	gtgcggccgcaagcttgtcgacggagctaaatatctcatcttcaag ataagtataaccgtac	

Table S3. List of primers used in this study.

References:

[1] Hamna, K.; Hamana, H.; Itoh, T. Ubiquitous Occurrence of Agmatine as the Major Polyamine within Extremely Halophilic Archaebacteria. *J. Gen. Appl. Microbiol.*, **1995**, *41*, 153–158.

[2] Tanaka T.; Hamana K.; Itoh I. Polyamine Analyses of Extermely Halophilic Archaebacteria and Methanogenic archaebacteria. *Ann. Gunma. Health.Sci*, **2002**, *23*, 137-143.

[3] Hamana, K.; Tanaka, T.; Hosoya, R.; Niitsu, M.; Itoh, T. Cellular Polyarnines of the Acidophilic, Thermophilic and Thermoack Dophilic Archaebacteria, Acidilobus, Ferroplasma, Pyrobacuium, Pyrococcus, Staphylothermus, Thermococcus, Thermodiscus and Vulcanisaeta. *J. Gen. Appl. Microbiol.*, **2003**, *49*, 287–293.

[4] Hamana, K.; Hosoya, R.; Itoh, T. Polyamine Analysis of Methanogens, Thermophiles and Extreme Halophiles Belonging to the Domain Archaea. *J. Japanese Soc. Extrem.*, **2007**, *6*, 25–31.

[5] Scherer, P.; Kneifel, H. Distribution of Polyamines in Methanogenic Bacteria. *J. Bacteriol.*, **1983**, *154*, 1315–1322.

[6] Allers, T.; Ngo, H.P.; Mevarech, M.; Lloyd, R.G. Development of Additional Selectable Markers for the Halophilic Archaeon Haloferax Volcanii Based on the leuB and trpA Genes. *Appl. Environ. Microbiol.*, **2004**, *70*, 943–953.

[7] Kaczowka, S.J.; Maupin-Furlow, J.A. Subunit Topology of Two 20S Proteasomes from Haloferax Volcanii. *J. Bacteriol.*, **2003**, *185*, 165–174.

[8] Blaby, I.K.; Majumder, M.; Chatterjee, K.; Jana, S.; Grosjean, H.; de Crécy-Lagard, V.; Gupta, R. Pseudouridine Formation in Archaeal RNAs: The Case of Haloferax Volcanii. *RNA*, **2011**, *17*, 1367–1380.

[9] Miyazaki, K. MEGAWHOP Cloning: A Method of Creating Random Mutagenesis Libraries via Megaprimer PCR of Whole Plasmids. *Methods Enzymol.*, **2011**, *498*, 399–406.

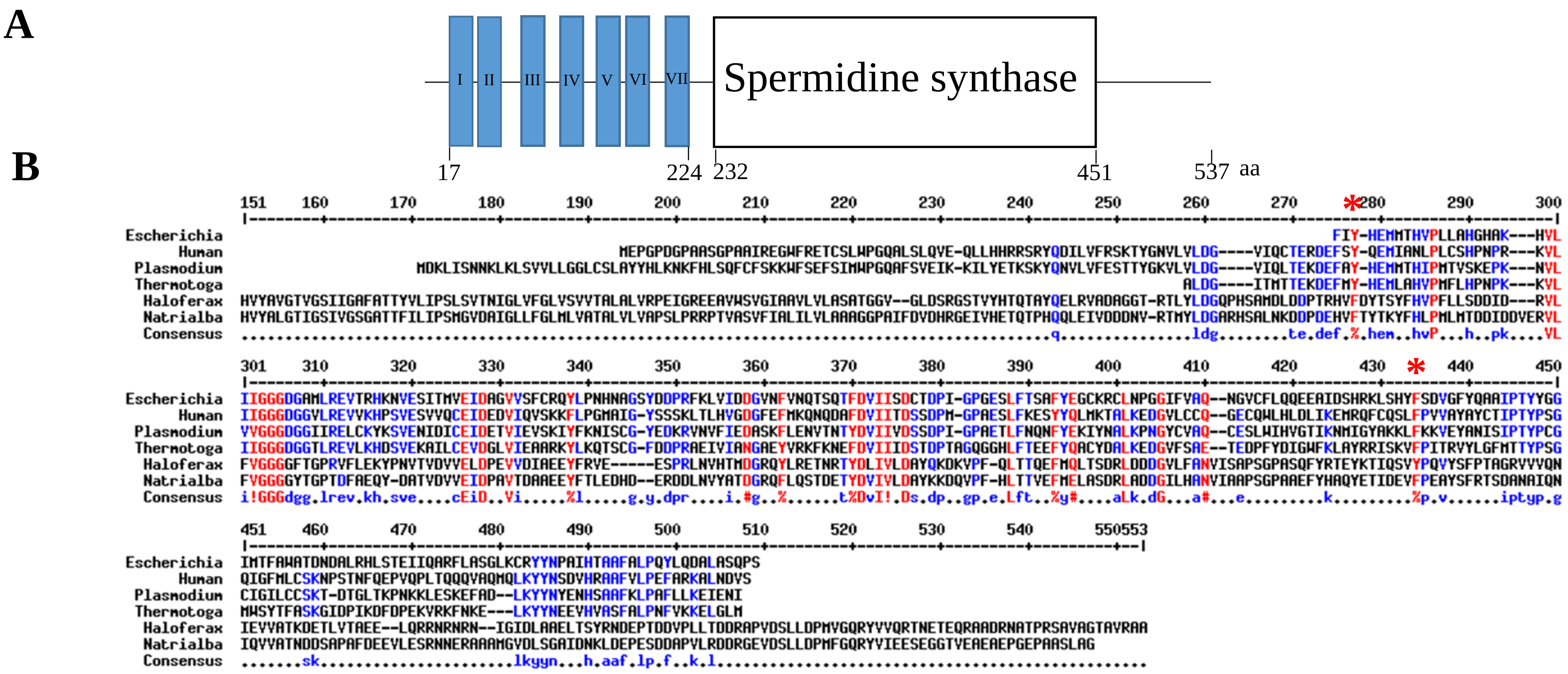

Fig. S1: Halophile spermidine synthases contain transmembranaire regions. *A*, prediction of transmembrane regions of HVO_0225 using TMHMM server v. 2.0. Transmembrane regions are indicated by roman numeral. I starts at amino acid 17 and end at 39; II starts at amino acid 44 and end at 66; III starts at amino acid 78 and end at 100; IV starts at amino acid 115 and end at 137; V starts at amino acid 150 and end at 172; VI starts at amino acid 177 and end at 196; VII starts at amino acid 205 and end at 224. NMAG_0842 from *Natrialba magadii ATCC 43099*, HTUR_3004 from *Haloterrigena turkmenica DSM 5511*; HALXA_2191 from *Halopiger xanaduensis SH-6*; HLRTI_13355 and HLRTI_11950 from *Halorhabdus tiamatea SARL4B* contain six to seven transmembrane domains. *B*, Multiple alignment of spermidine synthases using MultAlin (http://multalin.toulouse.inra.fr/multalin/). P09158 is *E.coli* SpeE; P19623 is Human SpeE; Q9WZC2 is *Thermotoga maritima* SpeE; D4GZK0 is *Hfx. volcanii* speE (HVO_0255); D3T068 is *Natrialba magadii* speE. Strictly conserved residues are in red; similar or partially conserved residues are in blue.

*putrescine binding site in human SpeE.

Supplemental S1

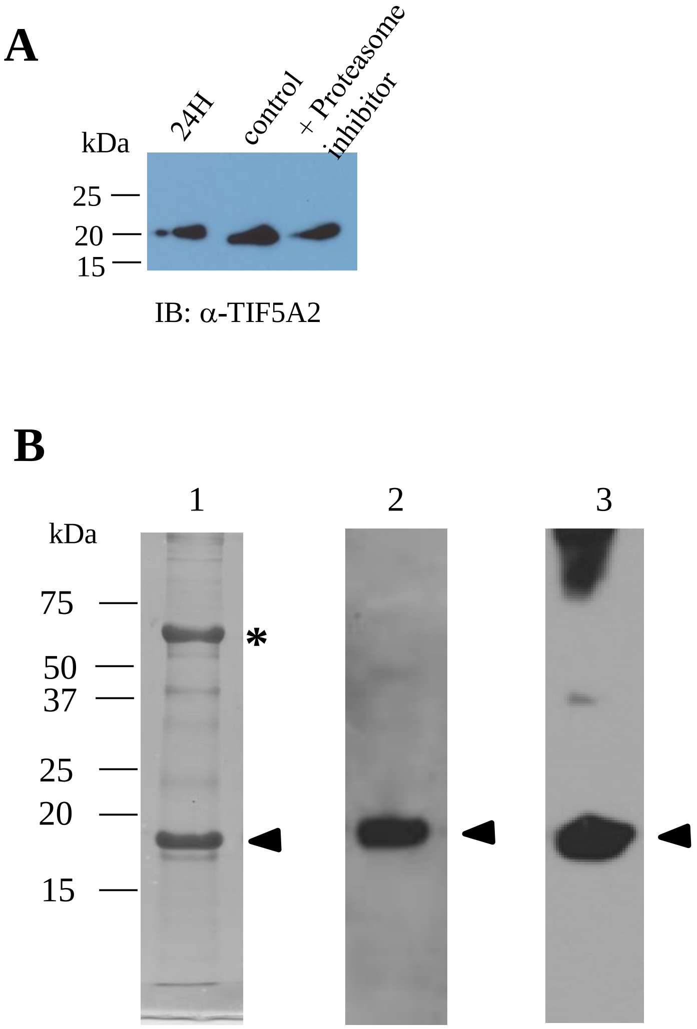

Fig, S2: No effect of proteasome inhibitor on aIF5A level and purified aIF5A is modified.

A, Cells were grown for 24 hours and then treated or not treated for 24H with 100 μM final bortozeomid (proteasome inhibitor). Equivalent protein loading was based on OD600 of cell culture (0.086 OD600 units per lane). Proteins were separated by 4-15% reducing SDS-PAGE. aIF5A was detected via α-aIF5A (anti-TIF5A2) immunoblot (IB). The molecular mass indicated are in kDa. B, Purified aIF5A (500 μl) was mixed with trichloroacetic acid for protein precipitation as described by Sanchez (Sanchez, 2011). After mixing with loading buffer and boiling, proteins were separated by reducing SDS-PAGE 12%. *Lane 1*, the proteins were detected by staining with Coomassie Blue R-250. *Lane 2*, aIF5A was detected by Western blotting raised against C-term His-tag. *Lane 3*, the deoxyhypusine/ hypusine modification was revealed by Western blotting raised against the deoxyhypusine/ hypusine modification (anti UI-88 antibody). Arrowhead indicates aIF5A; * a non-specific protein (pitA). Molecular mass markers are indicated in kDa.

Supplemental S2

A

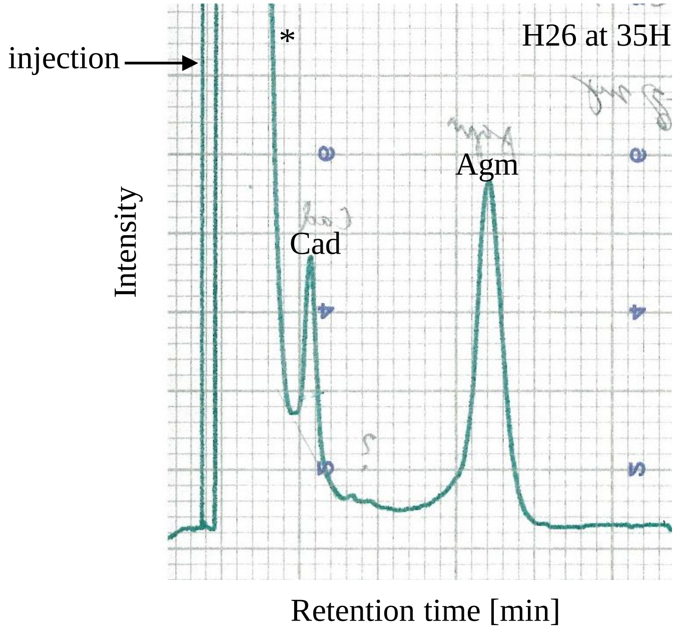

B

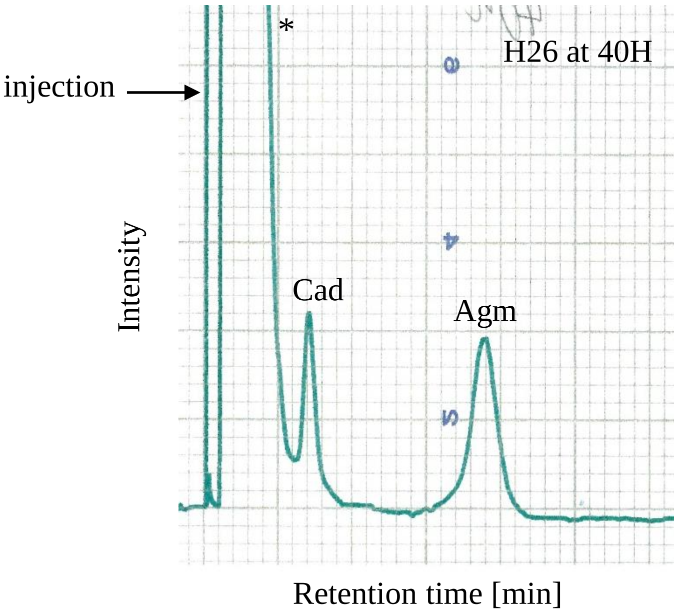

Supplemental S3

Fig. S3 : Intracellular polyamines analyzes in *Hfx. volcanii* H26 at 35H and 40H.

Intracellular polyamines from *Hfx. volcanii* H26 were extracted at different time of the growth. The cells were grown at 42° C in HV_min medium as described in the Materials and Methods section. *A*, samples after 35H of growth. Fifty seven mg of extracts were injected. The ratio agmatine/cadaverine is 12.81±10.41. B, samples after 40H of growth. Twenty six mg of extracts were injected. The ratio agmatine/cadaverine is 6.93 ±5.29. The injection is indicated by the arrow; *, unexpected noise derived from buffer. Standards are shown Fig 4A .

A

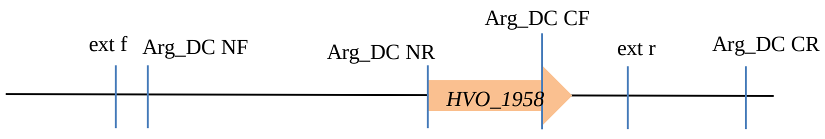

HVO_1958 plus 1 kb flank (2471 bp)

B

Region to amplify	Size of the PCR product
External mutant	1081 bp
External WT	1408 bp

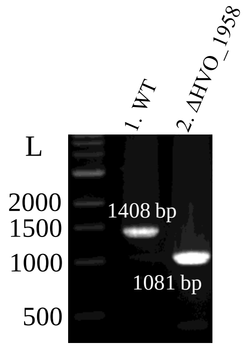

External

C

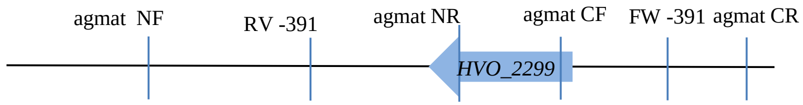

HVO_2299 plus 1 kb flank (2816 bp)

D

Region to amplify	Size of the PCR product
External mutant	1610 bp
External WT	922 bp

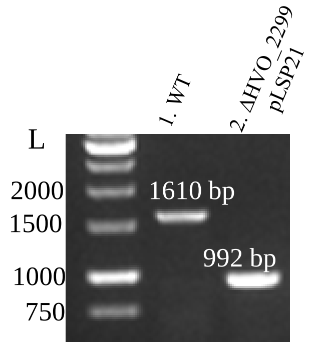

External

Fig. S4: Generation of *Hfx.volcanii* deletion constructions. *A*, Representation of *HVO_1958* (orange arrow) in the genome of the WT. The positions of primers used for the generation of the strain and to check the deletion are marked (summarized in Table S3). *B*, gene deletion of *HVO_1958* (VDC 3253) was verified by locus-specific PCR using primers listed in Table S3 designed to anneal outside the gene or inside. Amplicons size are indicated. L, ladder (NEB, 1 kb ladder); 1, amplicon from WT; 2, amplicon from Δ*HVO_1958*; bp, base pair. *C*, Representation of *HVO_2299* (blue arrow) in the genome of the WT. The positions of primers used for the generation of the strains and to check the deletion are marked (summarized in Table S3). *D*, gene deletion of *HVO_2299* in LSP5061 was verified by locus-specific PCR using primers listed in Table S3 designed to anneal outside the gene. Amplicons size are indicated. The band amplified in the Δ*HVO_2299* pLSP21 (LSP5061) was gel extracted and sequenced. L, ladder (gene ruler 1 kb, Thermo scientific); 1, amplicon from WT; 2, amplicon from Δ*HVO_2299* transformed with the plasmid pLSP21 (LSP5061); bp, base pair.

Supplemental S4

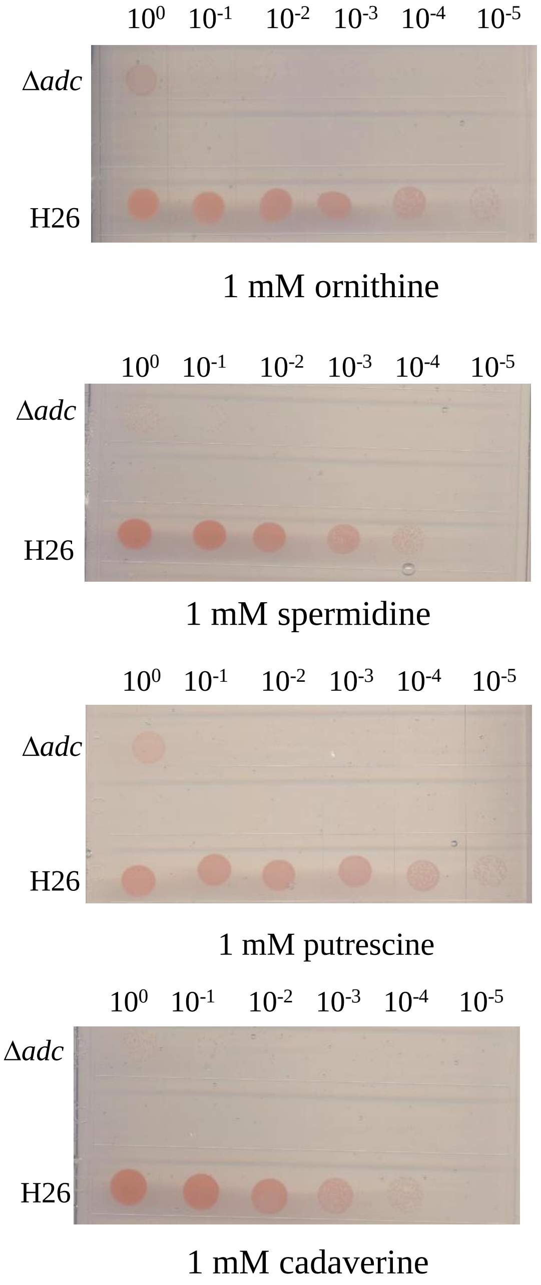

Fig. S5: Effect of different polyamines on the growth of Δ*adc*.

Hfx. volcanii H26 (WT, parent) and Δ*adc* were diluted to an OD_{600} of 1 and serial dilutions spot-plated (15 μl) on solid agar Hv_minimum medium as indicated. Each experiments was performed with two biological replicates and three technical replicates (see methods for description of biological vs. technical replicates).

Supplemental S5

A

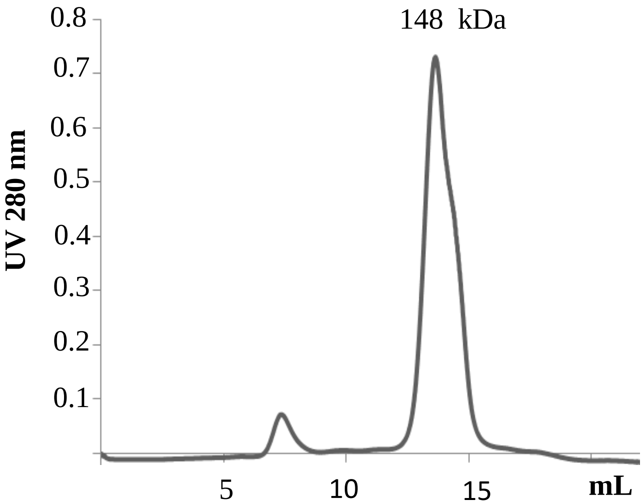

B

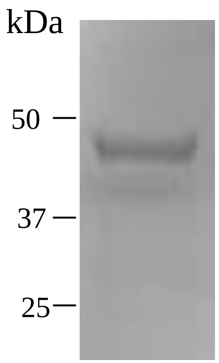

Fig. S6: PurifiedT7 His DHS from *Hfx. volcanii*.

T7-His-DHS purified as a tetramer complex of 148 kDa. *A*, superdex 200 (10/300 GL) chromatography of T7-HIS-DHS complex. B, 4 mg of T7 His DHS were separated by reducing SDS-PAGE 12% was detected by staining with Coomassie blue-R250. Molecular mass markers are indicated in kDa.

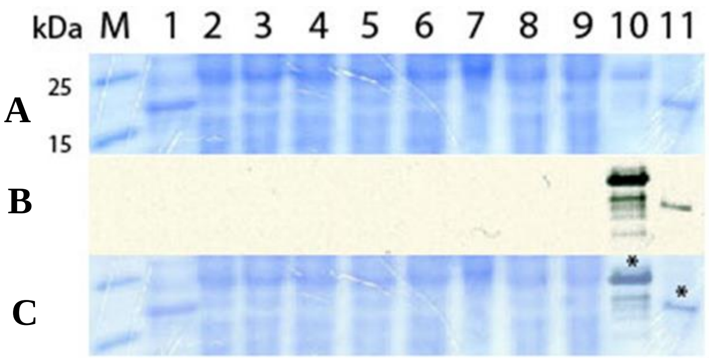

1	2	3	4	5	6	7	8	9	10	11	
+	+	+	+	+	+	+	+	+	-	+	aIF5A
-	-	-	-	-	-	-	-	-	+	-	eIF5A
-	+	+	-	-	+	+	-	-	-	-	aDHS
-	-	-	-	-	-	-	-	-	+	+	sDHS
-	+	-	+	-	-	-	-	-	+	+	[hot]Sperm
-	-	-	-	-	+	-	+	-	-	-	[hot]Putr
-	-	+	-	+	-	-	-	-	-	-	[cold]Sperm
-	-	-	-	-	-	+	-	+	-	-	[cold]Putr

Fig. S7: Deoxyhypusine synthase assay of *Hfx. volcanii*.

Detection of *Hfx.volcanii* aIF5A (lane 1, 2, 3, 4, 6, 7, 8, 11) and *S. cerevisiae* eIF5A (lane 10) modification using [^{14}C] spermidine (lane 2, 4, 10, 11) [^{3}H] putrescine (6, 8), non radioactive spermidine (3, 5) or non radioactive putrescine (7, 9) as substrates in presence of *Hfx. volcanii* DHS (lane 2, 3, 6, 7) or *S. cerevisiae* DHS (lane 10, 11). The *in vitro* assay was resolved on a 16.5 % tricine polyacrylamide gel. The dried gels were exposed to autoradiography films to visualize possible modifications. The assembly of each reaction is depicted above. *A*, Coomassie Blue staining. *B*, Exposed autoradiography film. *C*, Overlay of the Coomassie Blue stained gel and the exposed film. sDHS, Deoxhypusone synthase of *S. cerevisae*; aDHS, deoxyhypusine synthase of *Hfx. volcanii*.

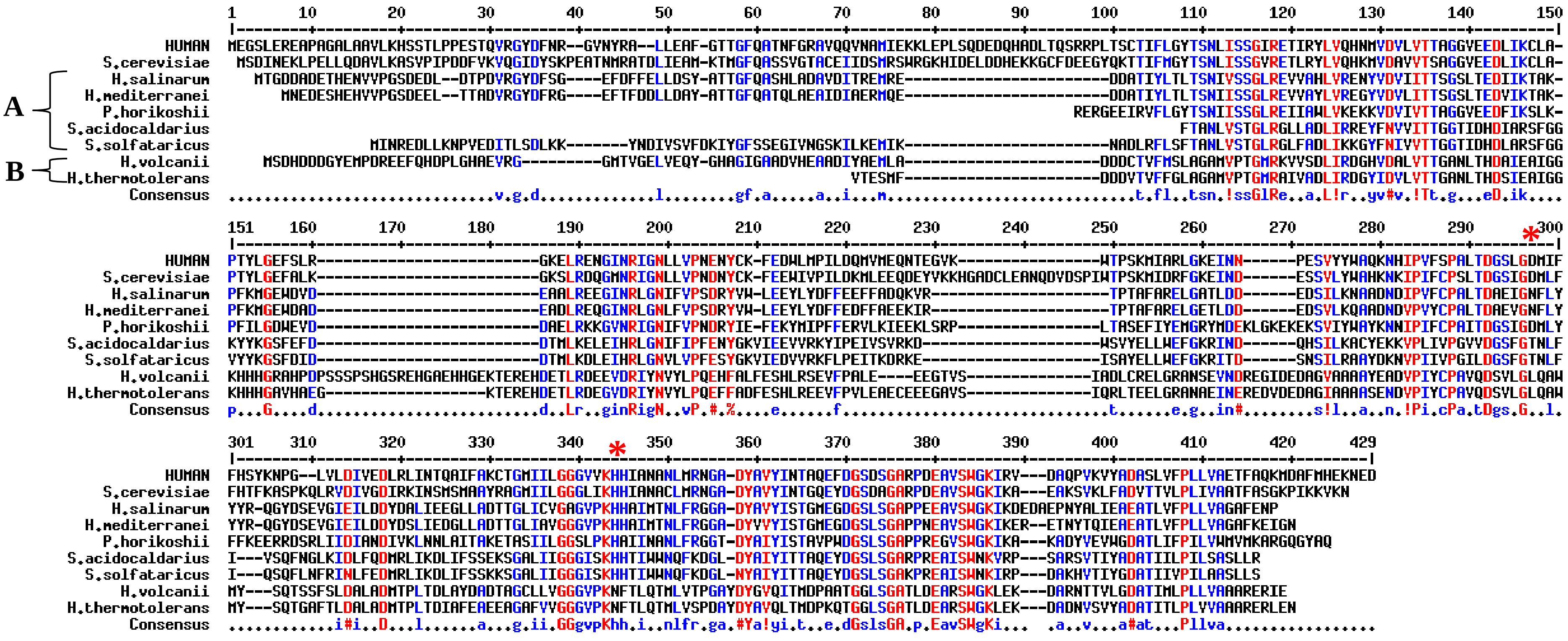

Fig. S8: Multiple alignment of deoxyhypusine synthase.

The alignment of DHS was performed using MultAlin (http://multalin.toulouse.inra.fr/multalin/). P49366 is Human DHS; P38791 *is Saccharomyces cerevisiae*; O50105 is *Pyrococcus horikoshii*; Q9HPX2IH is *Halobacterium salinarium* (Halobacterium halobium); I3RA05 is *Haloferax mediterranei*; Q4J978 is *Sulfolobus acidocaldarius*; Q97ZF1 is *Sulfolobus solfataricus*; D4GW0IH is *Haloferax volcanii*; M0C7L4IH is *Haloterrigena thermotolerans*. Strictly conserved residues are in red; similar or partially conserved residues are in blue. *spermidine binding site in human DHS and *S. cerevisae* DHS. *A*, DHS sequences from Archaea that are sensitive to GC7; *B*, DHS sequences from halophiles that harbor cadaverine .

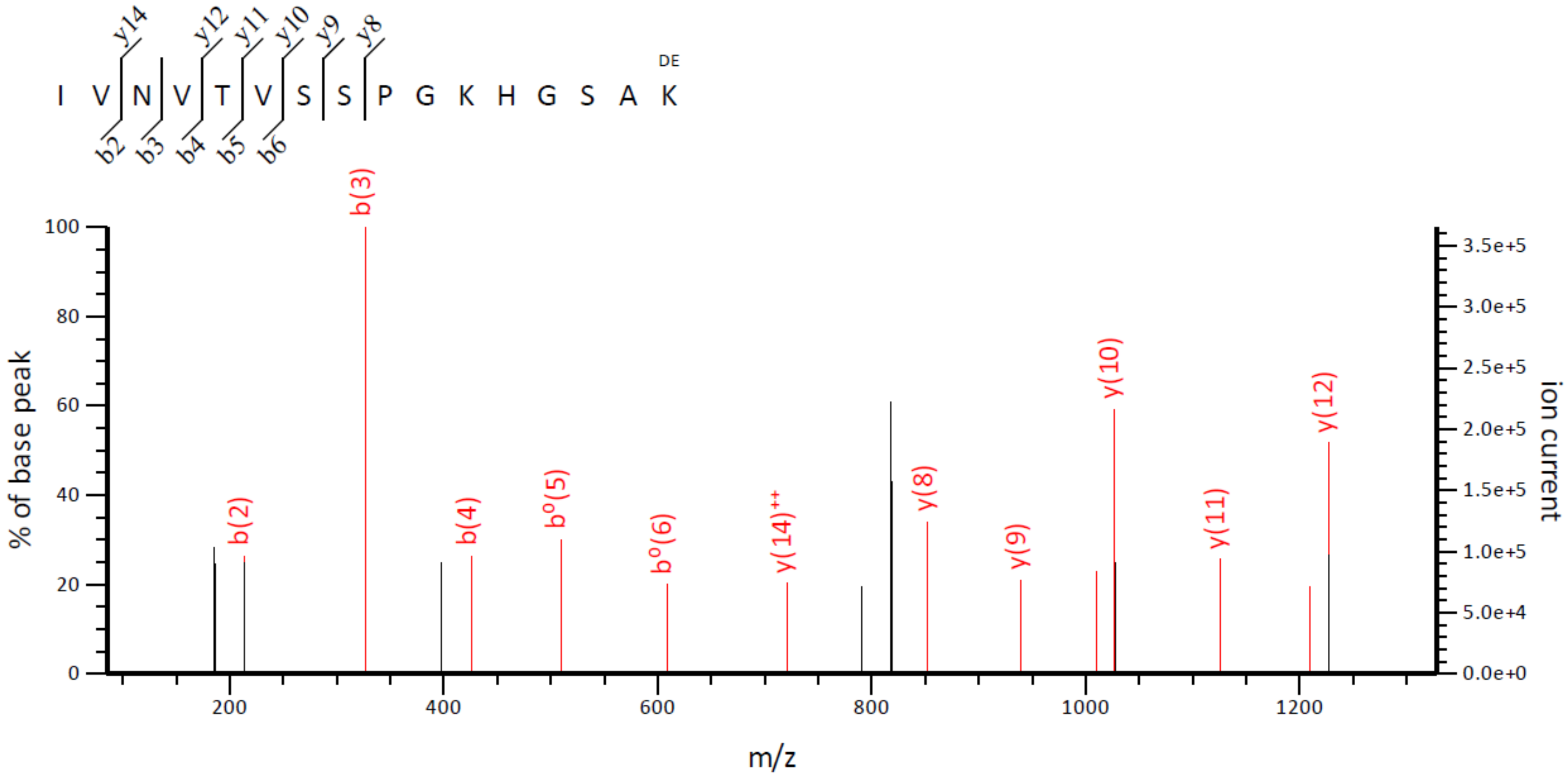

Fig. S9: Identification of *T. kodakarensis* aIF5A deoxhypusination by mass spectrometry.

50 µL of *in vitro* modification sample containing *T. kodakarensis* DHS (2.5 µM) and aIF5A (5 µM) were incubated in 0.2 M Glycine/NaOH (pH 9.4), 2 mM NAD+ and 50 µM spermidine at 30°C for 2 hours. Subsequently, the samples were analyzed using mass-spectrometry without further treatment. Fragmentation spectrum for deoxyhypusinylated peptide IVNVTVSSPGKHGSAK is shown, xiSPEC (http://spectrumviewer.org/) was used for annotation. DE, deoxyhypusine.
